# Systems biology of industrial oxytetracycline production in *Streptomyces rimosus*: the secrets of a mutagenized hyperproducer

**DOI:** 10.1186/s12934-023-02215-x

**Published:** 2023-10-28

**Authors:** Selma Beganovic, Christian Rückert-Reed, Hilda Sucipto, Wei Shu, Lars Gläser, Thomas Patschkowski, Ben Struck, Jörn Kalinowski, Andriy Luzhetskyy, Christoph Wittmann

**Affiliations:** 1 Institute of Systems Biotechnology, Saarland University, Campus A1 5, 66123 Saarbrücken, Germany; 2 Centre for Biotechnology, Bielefeld University, Bielefeld, Germany; 3 Department of Pharmacy, Saarland University, Saarbrücken, Germany

**Keywords:** Oxytetracycline, Systems biology, Streptomyces, Genome, Transcriptome, Proteome, Metabolome, Multiomics, Genetic instability, Mutagenesis

## Abstract

**Background:**

Oxytetracycline which is derived from *Streptomyces rimosus*, inhibits a wide range of bacteria and is industrially important. The underlying biosynthetic processes are complex and hinder rational engineering, so industrial manufacturing currently relies on classical mutants for production. While the biochemistry underlying oxytetracycline synthesis is known to involve polyketide synthase, hyperproducing strains of *S. rimosus* have not been extensively studied, limiting our knowledge on fundamental mechanisms that drive production.

**Results:**

In this study, a multiomics analysis of *S. rimosus* is performed and wild-type and hyperproducing strains are compared. Insights into the metabolic and regulatory networks driving oxytetracycline formation were obtained. The overproducer exhibited increased acetyl-CoA and malonyl CoA supply, upregulated oxytetracycline biosynthesis, reduced competing byproduct formation, and streamlined morphology. These features were used to synthesize bhimamycin, an antibiotic, and a novel microbial chassis strain was created. A cluster deletion derivative showed enhanced bhimamycin production.

**Conclusions:**

This study suggests that the precursor supply should be globally increased to further increase the expression of the oxytetracycline cluster while maintaining the natural cluster sequence. The mutagenized hyperproducer *S. rimosus* HP126 exhibited numerous mutations, including large genomic rearrangements, due to natural genetic instability, and single nucleotide changes. More complex mutations were found than those typically observed in mutagenized bacteria, impacting gene expression, and complicating rational engineering. Overall, the approach revealed key traits influencing oxytetracycline production in *S. rimosus*, suggesting that similar studies for other antibiotics could uncover general mechanisms to improve production.

**Supplementary Information:**

The online version contains supplementary material available at 10.1186/s12934-023-02215-x.

## Background

*Streptomyces* strains synthetize a rich spectrum of natural products, including more than 75% of all industrial antibiotics [1]. Because microbes naturally accumulate the molecules at low levels, improving the synthesis process is the cornerstone of commercial production [2]. In most cases, this process determines the overall economics. Typically, the biosynthesis of antibiotics is highly complex [3, 4], which makes the production of strains through rational metabolic engineering challenging [5]. Accordingly, strains are largely mutagenized and selected for improved performance [6], and the antibiotic manufacturing industry strongly maintains the use of these *classical mutants* for fermentative production [7]. These overproducers are powerful workhorses, although the mechanism underlying their efficiency is often unknown. This is also true for mutagenized strains of *S. rimosus,* which are used for commercial manufacturing of oxytetracycline, one of the most prominent antibiotics today [8].

Initially, the polyketide was first isolated from culture samples of *Streptomyces rimosus* and called terramycin [9]. The compound inhibits pathogenic gram-positive and gram-negative bacteria, making it a broad-spectrum antibiotic in the tetracycline family [10] and among the most often prescribed antibiotics [11]. Industrially, more than 20 suppliers manufacture oxytetracycline, producing over 10,000 tons [12], and according to recent estimates, prices have stabilized at approximately 20 US-$ kg^−1^. Furthermore, third-generation (semi)synthetic tetracyclines, such as tigecycline, demonstrate promising ability to surpass tetracycline-resistance mechanisms [8].

**Fig. 1:**
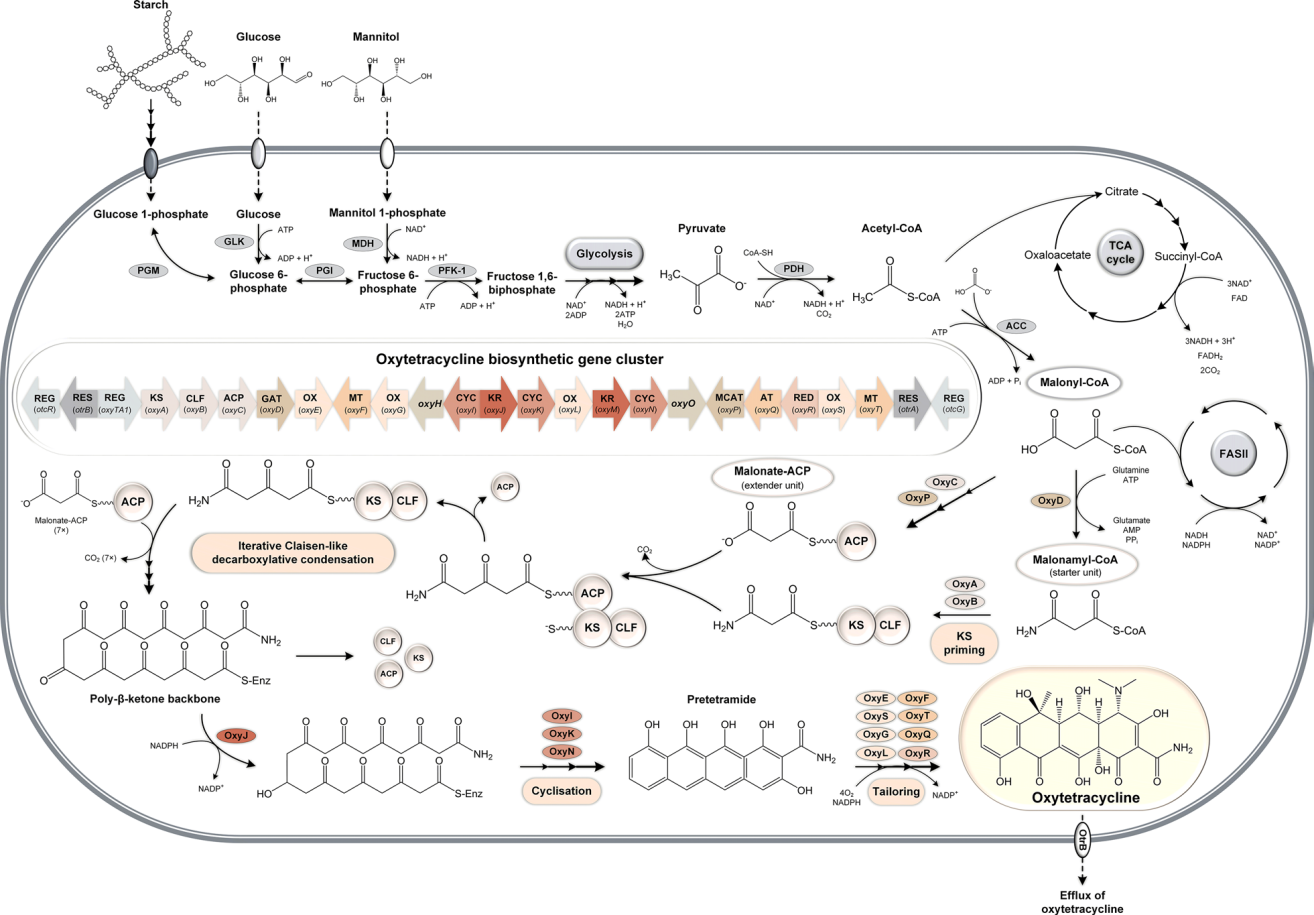
Biosynthesis of oxytetracycline in *Streptomyces rimosus*. Biosynthesis of the oxytetracycline backbone is catalysed by a minimal PKS through successive decarboxylative Claisen-like condensations of eight malonyl-CoA extender units to the amidated starter unit malonamyl-CoA [91]. Subsequently, the intermediate is reduced and modified by cyclases and aromatases, yielding an entirely aromatized intermediate called pretetramide [14]. Pretetramide undergoes multiple tailoring reactions catalysed by oxygenases, methyltransferases, aminotransferases, and reductases to finally generate the fully functional oxytetracycline, which is characterized by four aromatic rings and a C2-amide group and is specific for the tetracycline class of antibiotics. Abbreviations: TCA, tricarboxylic acid; FAS, fatty acid synthesis; PGM, phosphoglucomutase; GLK, glucokinase; PGI, phosphoglucose isomerase; MDH, mannitol dehydrogenase; PFK1, phosphofructokinase 1, PDH, pyruvate dehydrogenase; ACC, acetyl-CoA carboxylase; KS, ketosynthase; CLF, chain length factor; ACP, acyl carrier protein; GAT, glutamine amidotransferase; OX, oxygenase; MT, methyltransferase; KR,: ketoreductase; CYC, cyclase; ARO, aromatase; MCAT, malonyl-CoA:ACP acyltransferase; AT, aminotransferase; REG, regulation; RES, resistance. The function of the enzymes encoded by *oxyHO* is not yet fully understood

*S. rimosus* forms oxytetracycline by a type II polyketide synthase (PKS) [13] from nine molecules of malonyl-CoA, which is derived from acetyl-CoA via carboxylation [14] (Fig. 1). The underlying biochemistry has been well established [8]. The oxytetracycline gene cluster harbours genes for biosynthetic enzymes, transcriptional regulators, and genes that confer resistance [7, 15, 16]. Undoubtedly, mutagenized and oxytetracycline-overproducing *S. rimosus* strains are highly interesting to study but have not been extensively investigated thus far [17].

In this regard, the present work provides a multiomics view of *S. rimosus*. Using genomics, transcriptomics, proteomics, and metabolomics, we compared wild-type *S. rimosus* ATCC 10970 (R7) with the derivative *S. rimosus* HP126, which belongs to a family of classically derived hyperproducing strains [18]. The data provided deep insight into the underlying metabolic and regulatory networks that mediate high-level oxytetracycline formation. Strikingly, the overproducer utilized an activated supply of acetyl-CoA and malonyl CoA, oxytetracycline building blocks, upregulation of oxytetracycline biosynthesis, reduced pathway activities towards competing byproducts, and a streamlined morphology. Building on these traits, we evaluated its use for the heterologous synthesis of bhimamycin, a rare octaketide with antibiotic properties [19]. Furthermore, we created a novel microbial chassis strain. The strain *S. rimosus* HP126 Δv3, obtained from the producer by deletion of the cluster, also showed improved bhimamycin production, opening possibilities to streamline *S. rimosus* for the synthesis of novel heterologous polyketides.

## Results

The classical mutant *S. rimosus* HP126 accumulates up to 65-fold more oxytetracycline than that of its parent wild-type *S. rimosus* ATCC 10970. A central goal of this study was to unravel the molecular changes that occur during classical mutagenesis of *S. rimosus*. Hereby, the analysis of the microbe at the genome, transcriptome, proteome, and metabolome levels should provide a detailed picture, *inter alia* allowing us to uncover functional interactions between the different cellular components and improve our knowledge at the systems level [20, 21].

As a starting point, we evaluated the difference in oxytetracycline production between *S. rimosus* HP126, a previously obtained mutagenized derivative, and its parent strain *S. rimosus* ATCC 10970. To this end, we placed both strains in submerged batch cultures using a complex starch-containing medium similar to the industrial media used for decades to manufacture oxytetracycline [22]. Within 120 h, the wild type accumulated 70 mg L^−1^ of oxytetracycline, underlining the natural ability of *S. rimosus* to excrete the antibiotic. In comparison, strain HP126 produced 4,490 mg L^−1^ oxytetracycline (Additional file 1, Fig. S1A), 65-fold more than its ancestor.

**Fig. 2:**
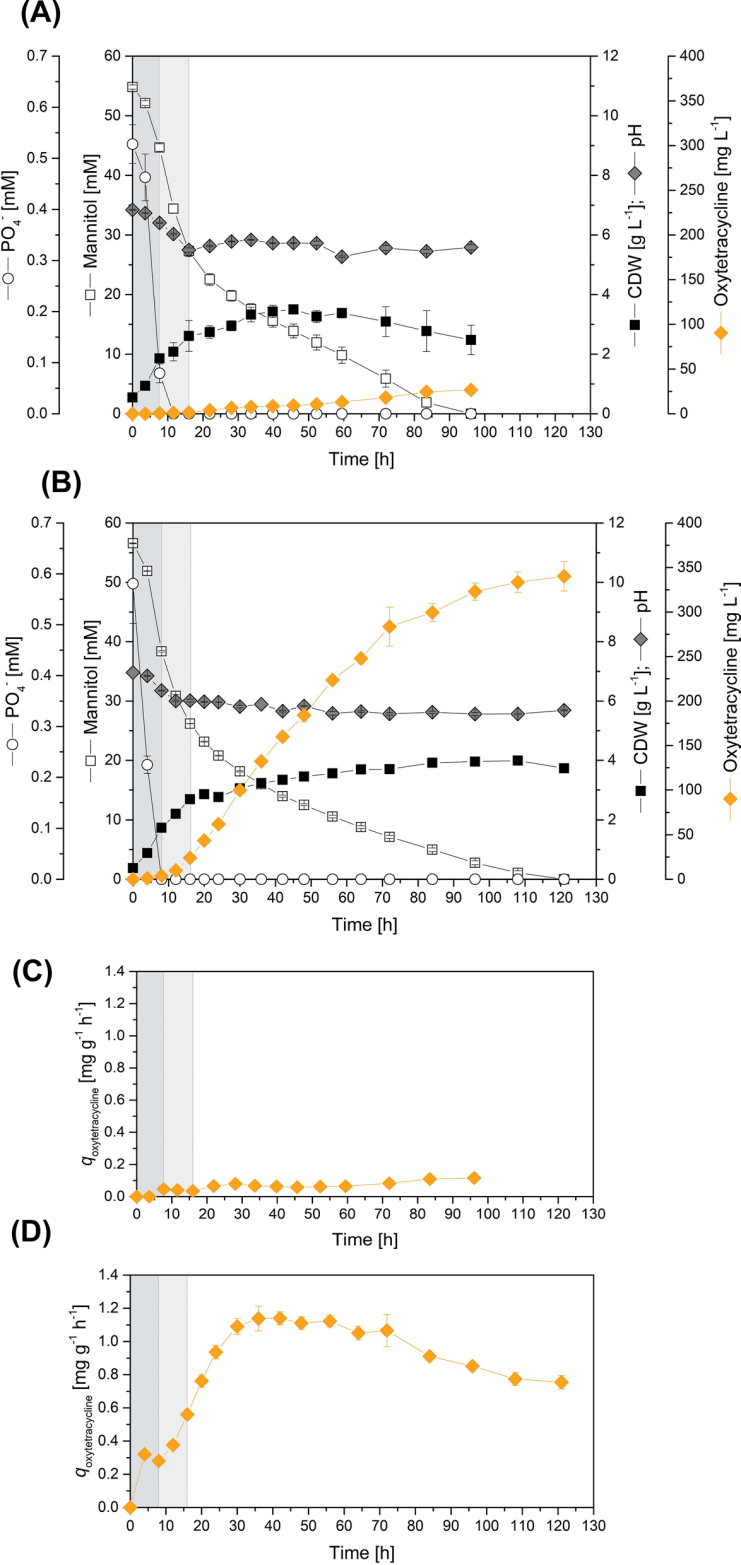
Growth and oxytetracycline production in wild-type *S. rimosus* ATCC 10970 (R7) and its hyperproducing mutagenized derivative HP126. The data comprise the fermentation profiles over time on mannitol-based minimal medium (**A**, **B**) and the resulting specific oxytetracycline production rate (**C**, **D**). The phases of (i) exponential growth without oxytetracycline production, (ii) reduced growth and production at the onset of phosphate limitation, and (iii) major oxytetracycline production during the stationary phase are indicated by vertical lines. n = 3

Next, we studied both strains on mannitol-based minimal medium, which we developed to facilitate the interpretation of the expected multiomics data [4, 23]. Mannitol was determined to be the optimal carbon source among a range of tested carbohydrates (Additional file 1, Fig. S1B). When the growth and production dynamics were monitored over time, *S. rimosus* ATCC 10970 went through the following culture phases: an initial phase of exponential growth with a maximum growth rate of *µ* = 0.16 h^−1^, a transition phase of reduced growth, and a final stationary phase (Fig. 2A). Growth slowed down after phosphate was depleted. The strain continued to utilize mannitol during the stationary phase at a constant rate. Mannitol was completely consumed after 96 h. Oxytetracycline accumulated almost linearly, resulting in a low but stable specific productivity (Fig. 2C). At the end of the process after 120 h, a final oxytetracycline titre of 27 mg L^−1^ was reached. The antibiotic was not detectable during the initial growth phase, but its production started once phosphate was limiting. The pH value decreased to pH 5.5 during the first 16 h, slightly increased again afterwards, and stabilized at approximately 6.0.

*Streptomyces rimosus* HP126 strongly differed in growth and oxytetracycline production (Fig. 2B). The mutant accumulated the antibiotic to a final level of 340 mg L^−1^, 12-fold more than the wild type. Furthermore, it grew faster during the initial phase (*µ* = 0.19 h^−1^) and exhibited an almost twofold higher specific phosphate uptake rate, which led to an earlier onset of phosphate limitation. Oxytetracycline was detectable after 4 h. Another difference from the wild type was the slower consumption of mannitol. The substrate was only consumed after 120 h, more than one day later. On the other hand, the biomass concentration was maintained until the end. The specific oxytetracycline productivity increased to a maximum value of 1.1 mg g^−1^ h^−1^ (Fig. 2D), remained at this high level over a period of up to 60–70 h, and finally slightly decreased (to 0.8 mg g^−1^ h^−1^). The chosen culture setup turned out to be suitable for the planned systems biology studies in terms of the following important aspects: (i) it allowed reproducible experiments, clearly visualized (ii) the phenotypic differences between the strains, and (iii) provided a stable oxytetracycline production phase so that, in each case, selected time points were representative of strain characteristics.

**Fig. 3:**
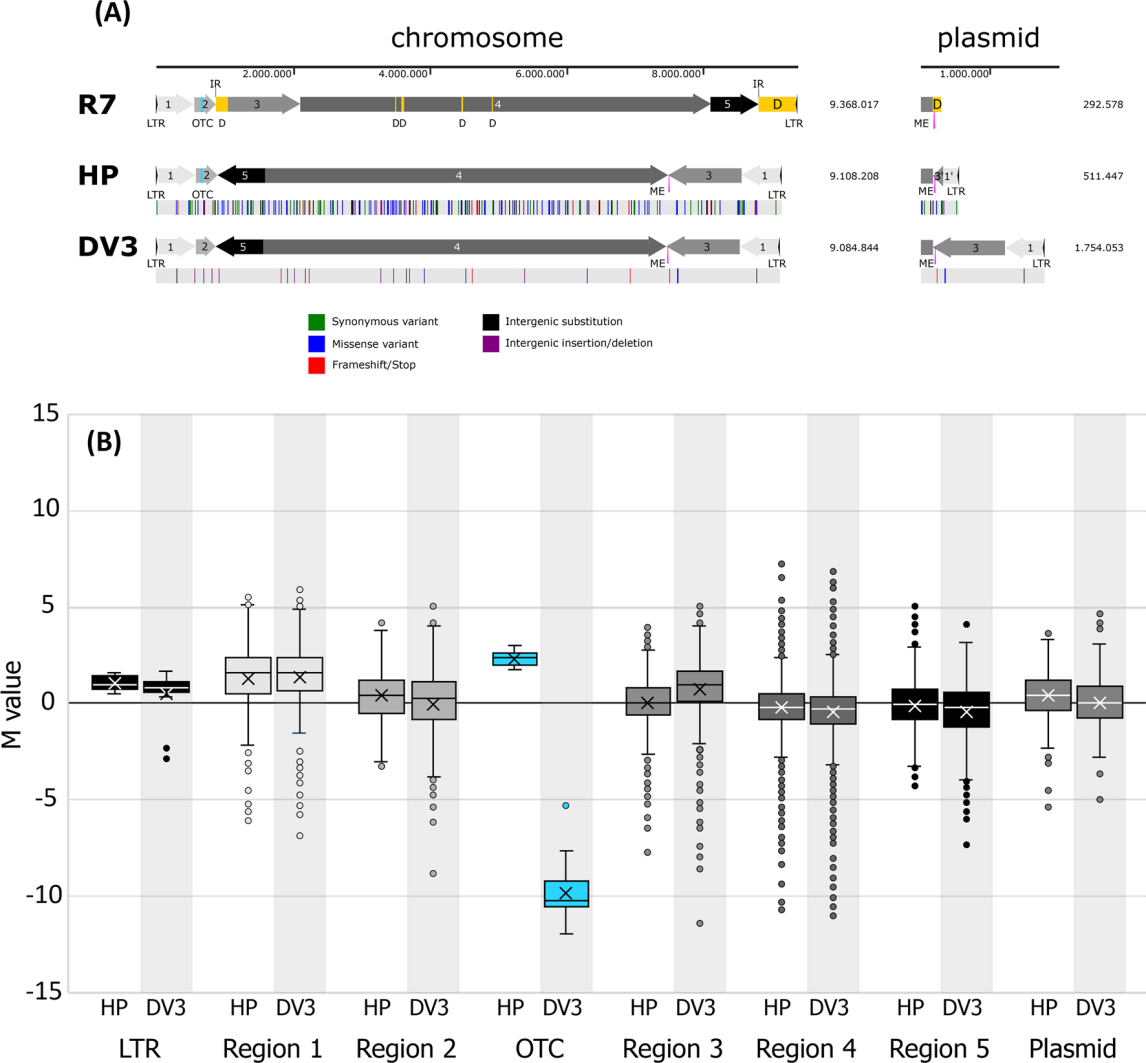
Genomic profiling of the wild type *S. rimosus* ATCC 10970 (R7), its mutagenized oxytetracycline-hyperproducing derivative HP126 (HP), and the chassis strain HP126 *∆v3* (DV3), created by deletion of the oxytetracycline cluster. The data show the genomic repertoire of the linear chromosome and the plasmid of the three strains (**A**) and the distribution of gene expression across the different genomic regions (**B**). The different chromosomal and plasmid-encoded segments are numbered from 1 to 5. These segments are shown as arrows to visualize their position and orientation in the wild type and their rearranged position and orientation in the mutants, as determined by BLASTN comparison. Deleted genomic regions that were lost during the mutagenesis process are shown in yellow. The oxytetracycline cluster is shown in light blue. Single nucleotide polymorphisms (SNPs) between the strains are indicated by colour. The gene expression data reflect the average expression of all genes that belong to a specific genomic region. The M-value reflects the difference in expression in strains HP and DV3 compared to the wild type. Green and red arrows highlight expression changes superimposed by genomic rearrangement events, such as multiplication and deletion. LTR, long terminal repeat; D, deletion; IR, inverted repeat; ME, mobile element

Genomic profiling of the mutagenized overproducer *S. rimosus* HP126 revealed 184 single nucleotide exchanges and massive genome rearrangements. The wild type strain R7 exhibited a linear chromosome of 9,368,017 bp with telomers that contained long terminal inverted repeats (LTRs) (Fig. 3A), representing the typical genomic layout of *Streptomyces* [24]. The chromosome contained 8,069 open reading frames. In addition, a large linear plasmid of an additional 292,576 bp with another 296 open reading frames was found. The oxytetracycline biosynthesis-related gene cluster was located on the chromosome, close to one of the telomers.

Notably, the overproducer exhibited large genome rearrangements (Fig. 3A). Its linear chromosome lacked 812.1 kb of sequence (8.67%) but gained 539.8 kb (5.76%) through the duplication of the left terminal region (designated region 1) and was thus almost 3% smaller than that of its ancestor. Several regions were found to be eliminated from the chromosome. The largest deletions (∆) occurred in the terminal 2 Mb regions of the chromosome, leading to a loss of 171.9 kb at the end of region 3, which is close to the oxytetracycline cluster, and 543.1 kb at the right end. In addition, four segments between 5.5 and 38.7 kb in the central core (designated region 4), which are known to contain most of the essential genes in *Streptomyces* [25], were also eliminated. Overall, the deletions resulted in a loss of 619 genes from the terminal regions and 100 genes from the middle core.

Moreover, the position and orientation of two large sequence blocks that were 1,061.4 kb (shortened region 3) and 700.2 kb in length (complete region 5) had changed. Finally, the left terminal chromosomal region (550.7 kb) was found to be duplicated and inverted at the new right chromosome end. The linear plasmid was also structurally affected. It now comprised 511,447 bps, almost 60% more than that of the wild type. While one third of the original wild-type sequence had disappeared (124.0 kb), the mutant plasmid had acquired approximately 199.9 kb (36.3%) and 127.4 kb (12.0%) by large-scale duplications from chromosomal regions 1 and 3, respectively. The mechanism(s) involved in these large-scale genomic rearrangements remain to be determined, but at least for two events, homologous recombination can be inferred as part of the process: directly upstream of the plasmid region lost in strain HP126, a mobile element (ME) that also carried phage integrase was present on the plasmid in strain R7, a second copy of which was also found between regions 4 and 3 in strain HP126. Closer inspection of the BLASTN results also revealed the presence of an inverted repeat (IR) immediately downstream of region 2 and downstream of region 5. Both, the ME and the IR might have provided the substrate for homologous recombination events. Regardless of the cause, various genes from regions 1 (221) and 3 (225) were present as two copies as a result of the genomic rearrangements, whereas 182 genes from region 1 even occurred in the form of three copies in the mutant, and six copies existed for one gene (Additional file 2).

In addition, strain HP126 revealed 143 single nucleotide exchanges within coding genes in the chromosome, including 47 synonymous and 80 missense variants but also 16 mutations resulting in frameshifts and stops. In addition, 41 mutations were present in the intergenic sequence space, comprising 28 substitutions and 13 deletions or insertions. In total, 137 genes were subjected to mutations. The mutations were mainly located in core region 4, whereas the terminal regions carried significantly fewer SNPs. Within the core, a few hot spots with more SNPs could be observed. Surprisingly, no SNPs were found within the 44 genes of the oxytetracycline cluster. The plasmid also carried a substantial number of SNPs (Fig. 3A).

Global transcription in *S. rimosus* HP126 is strongly affected by mutagenesis-related genomic deletion and duplication events. To assess global gene expression, both strains were analysed by RNA sequencing. We selected a time point after 24 h for sampling, representing the phase of stable oxytetracycline production (Fig. 2). The expression of 2497 out of the 8534 genes (present in the wild type on the chromosome and the plasmid) was significantly altered in the hyperproducer (log2-fold change ≥ 1, p value ≤ 0.05). In this regard, 29.3% of the genomic repertoire was affected in expression, whereby 18.3% of the genes were downregulated and 11.0% of the genes were upregulated. Silenced expression logically resulted in genes that were lost during the mutagenesis process. Increased expression, on the other hand, was observed, especially for the duplicated and triplicated areas. For example, the average expression of genes belonging to the LTR region and to region 1 was significantly increased, whereby the resulting M-values nicely matched the change in gene copy number. This general region-specific trend superimposed the response in individual genes that were differentially reduced and increased in expression. The same effects were observed for plasmid-encoded genes. In contrast, the average gene expression was unaffected in other (not rearranged) genomic regions of the producer. Here, expression differences were restricted to selected changes in specific genes, which, however, were partially very strong (M-value >  + 5 and < -5). Taken together, the changed genomic repertoire of the producer exhibited a significant impact on global gene expression. The deletion and duplication events that had occurred and changed gene copy numbers contributed obviously strongly to this phenomenon.

**Fig. 4:**
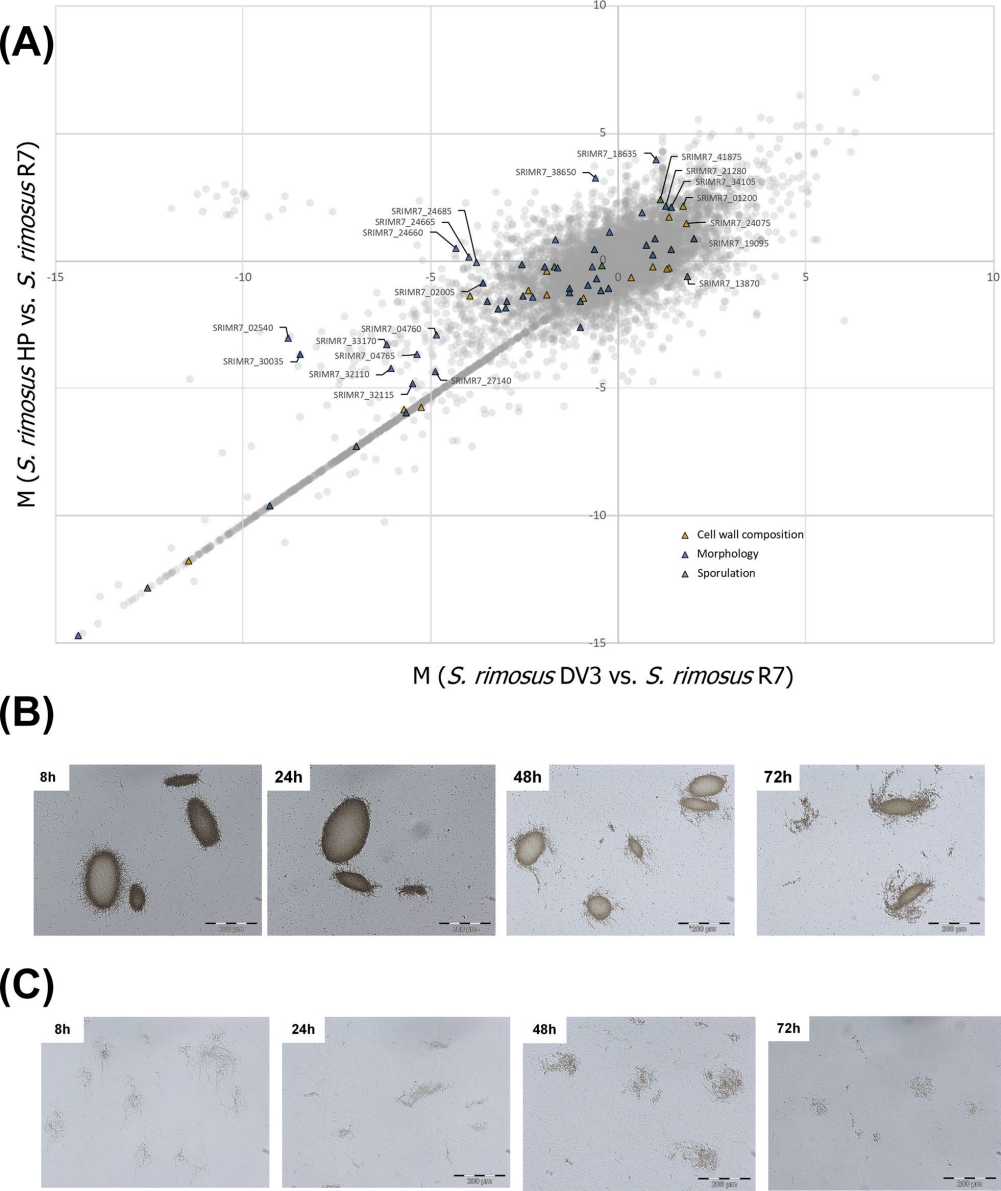
A systems view on morphological development of the wild type S. rimosus ATCC 10970 (R7), its mutagenized oxytetracycline-producing derivative HP126 (HP), and the chassis strain HP126 Δv3 (DV3), created by deletion of the oxytetracycline cluster. The data show differences in the expression of genes associated with morphology, sporulation, and cell wall biosynthesis (**A**) (n=3). The differences in gene expression are represented as M-values between strain HP versus R7 (y-axis) and strain DV3 versus wild-type (x-axis) so that correlations between all strains can be inferred from the plot. The data represent the major phase of oxytetracycline production after 24 h. The functional annotation of genes, designated by SRIMR7 numbers on basis of the sequenced genomes, is provided in Additional file 2. In addition, the data show changes in the cellular morphology, visualized by light microscopy at different time points of cultivation (8h, 24 h, 48 h, 72h), for the wildtype (**B**) and the hyper-producer HP126 (**C**)

A first glance into the gene expression of individual cellular processes revealed substantial changes related to morphological development, cell wall composition, and sporulation (Table 1, Fig. 4A, Additional file 3, Table S1). For example, the incorporation of teichoic acids (TAs) into the cell wall appeared to be impaired through downregulation of TA exporters. Likewise, the expression of genes encoding UDP-forming cellulose synthase, which is involved in the synthesis of the cellulose-like component of the extracellular matrix, was reduced, while the increased expression of genes encoding chitinases pointed to eventual cell wall degradation in *S. rimosus* HP126 by endogenous cell wall-degrading enzymes, which was also suggested by the enhanced expression of the murein DD-endopeptidases. Previously, cellular morphology was shown to influence oxytetracycline production in *S. rimosus* [26]. For a more detailed examination, we conducted microscopic studies. The wild type formed dense pellets with protruding filaments at the surface (Fig. 4B), which strongly differed in size. Pellet heterogeneity was assessed on a quantitative basis using software-supported estimation of the maximum Feret diameter, which represented the smallest circle into which a pellet could fit [3]. The analysis revealed that individual pellets differed up to tenfold (36 µm to 327 µm). During later culture stages, pellet ageing was observed. The aggregates started to fragment, linked to substantial disintegration of the biomass inside the structures. Notably, *S. rimosus* HP126 formed loose mycelial networks of hyphae instead, corresponding to a mat-type structure (Fig. 4C). Unlike the wild type, the aggregates did not exhibit decomposition effects over time.

**Table 1:** Expression of selected genes associated with cell metabolism and morphology

Locus tag	Annotation	Fold-change
*SRIMR7_14410*	Chitinase D	2.2
*SRIMR7_38545*	Poly-ß-1,6-N-acetylglucosamine synthase	1.8
*SRIMR7_12545*	Chitinase 63	1.7
*SRIMR7_24075*	Murein DD-endopeptidase, *mepM*	1.4
*SRIMR7_11730*	Exochitinase	1.3
*SRIMR7_01200*	Chitinase C	1.1
*SRIMR7_38525*	Chitinase D	1.1
*SRIMR7_01835*	Chitinase	1.1
*SRIMR7_25310*	Glycerophosphotransferase, *tagF*	−1.1
*SRIMR7_25990*	Cellulose synthase (UDP-forming)	−1.3
*SRIMR7_37765*	Chitinase class I	−1.3
*SRIMR7_25305*	Putative glycosyltransferase, *epsJ*	−1.4
*SRIMR7_38620*	Putative poly(glycerol-phosphate) alpha-glucosyltransferase, *tagE*	−1.5
*SRIMR7_25250*	Teichoic acid export, ATP binding protein, *tagH*	−5.7
*SRIMR7_25255*	Teichoic acid export, ATP binding protein, *tagG*	−5.8

The data are taken from the transcriptome analysis of *S. rimosus* ATCC 10970 and its mutagenized derivative HP126 (Fig. 2), sampled during the major production phase (24 h). n = 3

**Fig. 5:**
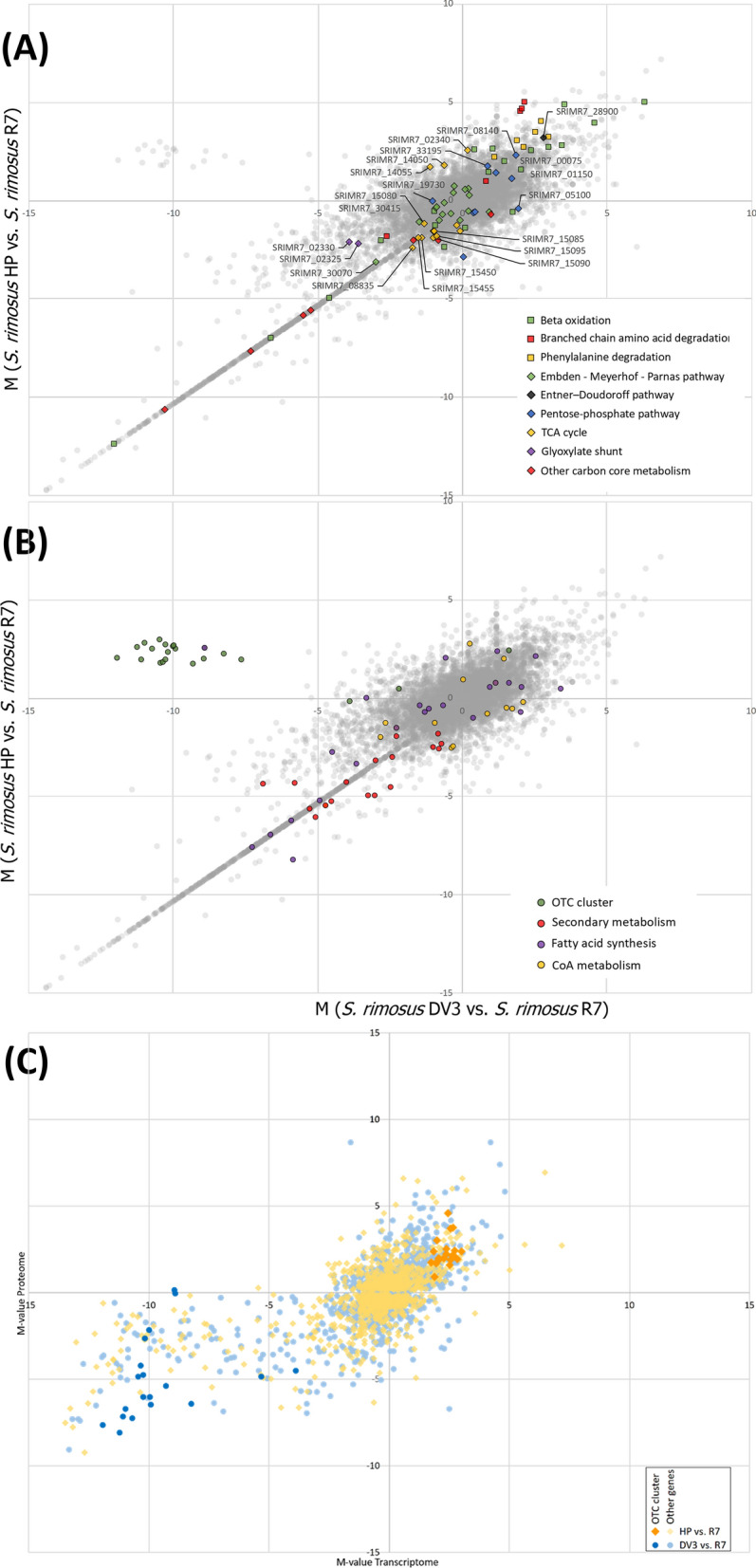
Transcriptomic and proteomic profiling of the wild type *S. rimosus* ATCC 10970 (R7), its mutagenized oxytetracycline-producing derivative HP126 (HP), and the chassis strain HP126 ∆*v3* (DV3), created by deletion of the oxytetracycline cluster. The data show differences in the expression of genes associated with primary catabolic and anabolic carbon metabolism (**A**) and secondary metabolism (**B**). The differences in expression are represented as M-values between strain HP versus R7 (y-axis) and strain DV3 versus wild-type (x-axis) so that correlations between all strains can be inferred from the plot. The data represent the major phase of oxytetracycline production after 24 h. The functional annotation of genes, designated by SRIMR7 numbers on basis of the sequenced genomes, is provided in Additional file 2. Correlation between gene expression and protein abundance in all strains after 24 h (**C**). The differences between strains are represented as M-values for the proteome (y-axis) and the transcriptome (x-axis). The oxytetracycline cluster genes are shown in orange (HP versus R7) and dark blue (DV3 versus R7). n = 3

Within primary carbon metabolism, most genes of the TCA cycle and the glyoxylate shunt were significantly downregulated (Fig. 5A). From a metabolic viewpoint, both pathways were fuelled from acetyl-CoA and thus competed with oxytetracycline biosynthesis, which also utilizes this precursor. If the reduced gene expression translates into reduced carbon flux into the TCA cycle and glyoxylate shunt, then the observed transcriptional changes are a first indicator that CoA-ester metabolism is possible disrupted.

Transcriptomic analysis reveals that expression of the oxytetracycline cluster in the mutant is activated and global changes occur to increase the supply of CoA-precursor. Next, we inspected the expression of the oxytetracycline biosynthesis-related gene cluster and metabolic pathways that either helped to form the antibiotic by supplying precursors, redox power, and energy or competed for these resources (Fig. 5B). In strain HP126, the oxytetracycline cluster was strongly upregulated (Additional file 3, Table S2). On average, the expression increase was fivefold (log_2_-fold change = 2.3), whereby the oxygenase gene *oxyQ* (*SRIMR7_02475*) exhibited the strongest activation. Because the cluster itself did not carry a single mutation, its transcriptional activation was apparently caused by other mechanisms, e. g. changes in regulators encoded outside of the cluster.

**Fig. 6:**
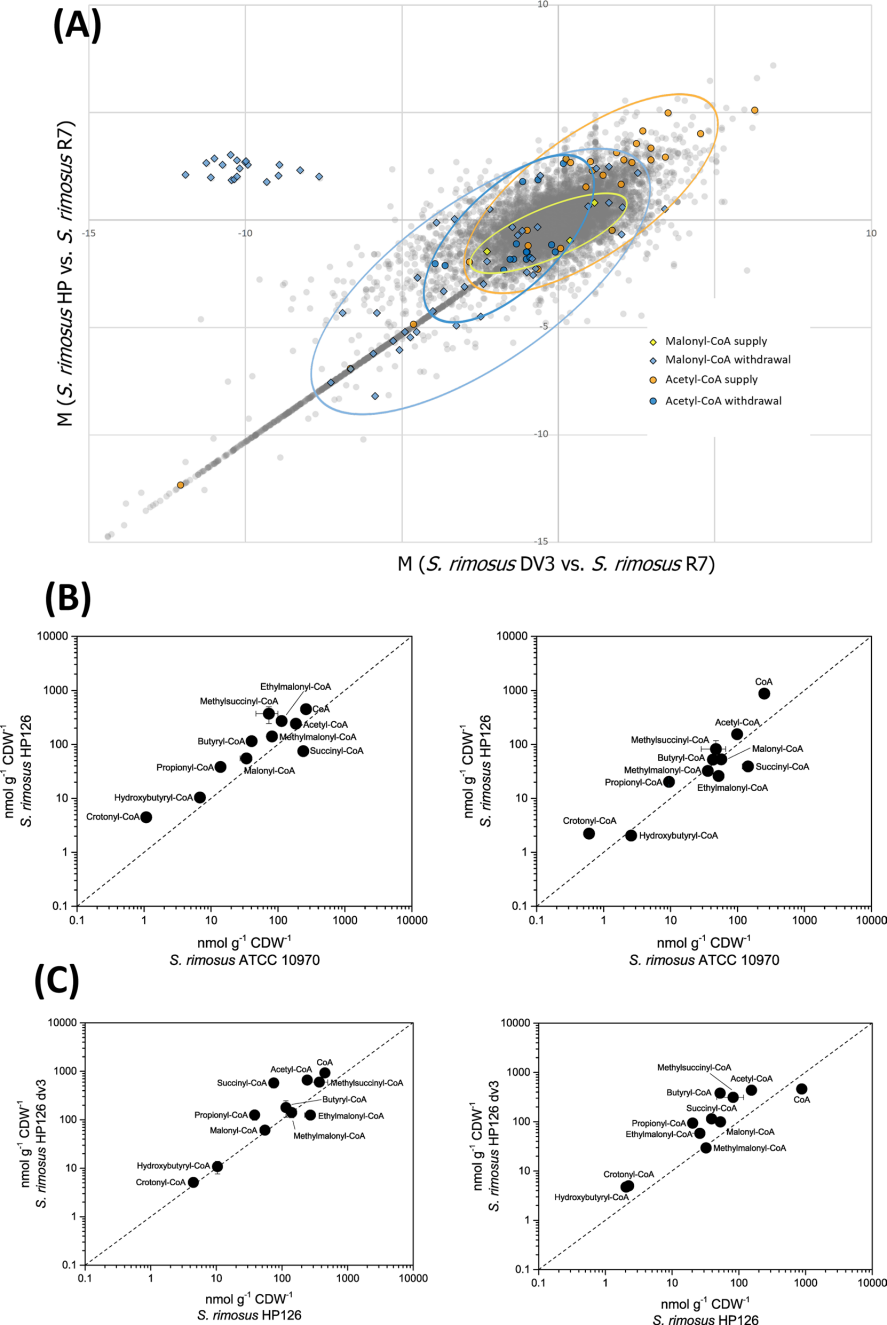
Integrated analysis of oxytetracycline production in *S. rimosus* at the transcriptome and metabolome levels. The studied strains comprise the wild type *S. rimosus* ATCC 10970 (R7), its mutagenized oxytetracycline-hyperproducing derivative HP126 (HP), and its non-producing counterpart HP126 ∆*v3* (DV3). Differences in the expression of genes associated with the biosynthesis and withdrawal of acetyl-CoA and malonyl-CoA during the major production phase (**A**). The differences are represented as M-values between strain HP versus R7 (y-axis) and strain DV3 versus R7 (x-axis) so that correlations between all strains can be inferred from the plot. Absolute intracellular levels of CoA thioesters (**B, C**). The metabolites were analysed in all strains at the following time points: 8 h (T1), 16 h (T2), 36 h (T3), and 50 h (T4), while the final sample (T5) was taken after 60 h (R7), 55 h (HP), and 72 h (DV3). The statistical significance of the observed differences in concentrations was estimated by a t-test: NS, not significant; *p* < 0.05, *; < 0.01, **). n = 3

Regarding pathways related to the supply and withdrawal of malonyl-CoA and its precursor acetyl-CoA, catabolic genes encoding acyl-CoA-forming enzymes were upregulated, including the branched-chain alpha-keto acid dehydrogenase complex, which is involved in the degradation of branched-chain amino acids (Table 2). Furthermore, the oxidative phenylacetate degradation pathway, yielding acetyl-CoA and succinyl-CoA, was upregulated. The same change was found for genes associated with lipid and fatty acid degradation (Additional file 3, Table S3). The expression of several lipases was also increased. Moreover, long-chain fatty acid CoA ligases (log_2_-fold change up to 5.0), acyl-CoA dehydrogenases, 3-hydroxyacyl-CoA dehydrogenases, and 3-ketoacyl-CoA thiolases exhibited increased expression. On the other hand, fatty acid anabolism was downregulated, including several 3-ketoacyl-ACP synthases engaged in initial condensation (log_2_-fold change up to -8.2). Finally, the biosynthetic routes to other CoA-ester-derived natural products were downregulated, including erythronolide and a nystatin-like compound (Fig. 5B, Additional file 3, Table S4). Taken together, genes encoding acetyl-CoA- and malonyl-CoA-forming enzymes tended to be upregulated, while genes encoding enzymes that withdrew these metabolites were predominantly downregulated (Fig. 6A). This observation indicated that a globally streamlined supply of the two oxytetracycline-building blocks is generated from the mutagenesis process.

**Table 2:** Expression of genes associated with CoA thioester metabolism

Locus tag	Annotation	HP126	HP126 Δv3
*SRIMR7_19995*	BC α-keto acid dehydrogenase E1 subunit α	5.0	−2.9
*SRIMR7_20005*	BC α-keto acid dehydrogenase E2	4.7	−2.7
*SRIMR7_20000*	BC α-keto acid dehydrogenase E1 subunit β	4.6	−2.7
*SRIMR7_19935*	1,2-Phenylacetyl-CoA epoxidase, subunit E	4.1	−1.4
*SRIMR7_19940*	1,2-Phenylacetyl-CoA epoxidase, subunit D	3.5	−1.0
*SRIMR7_19945*	1,2-Phenylacetyl-CoA epoxidase, subunit C	3.3	0
*SRIMR7_35410*	Phenylacetate-CoA ligase	3.1	−1.3
*SRIMR7_34430*	Coenzyme A biosynthesis protein CoaBC	2.9	0
*SRIMR7_11535*	Putative propionyl-CoA carboxylase β-chain	1.1	−2.6
*SRIMR7_11575*	BC amino-acid aminotransferase	1.0	−1.0
*SRIMR7_01685*	Crotonyl-CoA carboxylase/reductase	−6.4	0
*SRIMR7_07230*	Methylmalonyl-CoA mutase	−2.5	2.5
*SRIMR7_07225*	Crotonyl-CoA carboxylase/reductase	−2.4	2.4
*SRIMR7_21110*	Valine dehydrogenase	−1.7	0
*SRIMR7_02335*	3-Hydroxybutyryl-CoA dehydrogenase	−1.9	0
*SRIMR7_12355*	Methylmalonyl-CoA mutase	−1.2	−1.4
*SRIMR7_33660*	3-Hydroxybutyryl-CoA dehydrogenase	−1.2	1.2

The data are taken from the transcriptome analysis of *S. rimosus* ATCC 10970 and its mutagenized derivative HP126 (Fig. 2), which were sampled during the major production phase (24 h). The reference for *S. rimosus* HP126 was *S. rimosus* ATCC 10970, while the reference for *S. rimosus* HP126 *Δv3* was *S. rimosus* HP126. n = 3

Proteomic analysis revealed an increased abundance of oxytetracycline-synthetizing enzymes. Next, we analysed the proteome after 24 to investigate the correlation between protein and mRNA abundance. Among the proteins detected in all three biological replicates, 1,411 proteins were detected in the wild type and 1,322 proteins were detected in the producer, roughly representing 16.8% and 17.4% of the genomic repertoire, respectively (Fig. 5C). Overall, the proteome differed largely between the wild type and the oxytetracycline overproducer. The abundance of a significant number of proteins was increased or decreased, indicating that the protein inventory in the producer was largely changed. Interestingly, protein abundance and transcript abundance were significantly correlated for the oxytetracycline cluster. Increased expression of the cluster genes was reflected by a correspondingly strong increase in the abundance of the biosynthetic pathway enzymes (Fig. 5C). This observation was also found for many other genes, in which transcription strength and protein abundance correlated rather well. Regarding the formation of oxytetracycline, the increased abundance of the biosynthetic enzymes appeared to be an important driver for the enhanced formation of the antibiotic.

*S. rimosus* HP126 exhibits increased availability of intracellular CoA thioesters during the phase of oxytetracycline production. The various transcriptional changes suggested that the supply and withdrawal of CoA esters were disrupted (Fig. 6A). The impact of these alterations was assessed through absolute quantification of intracellular CoA-thioester pools. During the phase of high oxytetracycline production, the wild type contained ten different CoA-thioesters from carbon two to carbon five (Fig. 6B, left 16 h, right 34 h). Succinyl-CoA was the most abundant derivative, followed by acetyl-CoA, methylmalonyl-CoA, ethylmalonyl-CoA, and methylsuccinyl-CoA. Butyryl-CoA and malonyl-CoA were found in the mid-concentration range, while propionyl-CoA, hydroxybutyryl-CoA, and crotonyl-CoA were present in traces and represented less than 1% of the total spectrum. Qualitatively, the mutant contained the same derivatives, but most were increased in abundance (Fig. 6B). Regarding oxytetracycline formation, acetyl-CoA was increased at both time points. The malonyl-CoA pool was higher in the producer after 16 h, while after 34 h, both strains exhibited a similar level. Notably, the ratio between individual CoA-thioesters was also disrupted when comparing the two strains, e.g., this was observed for butyryl- and hydroxy-butyryl-CoA.

**Fig. 7:**
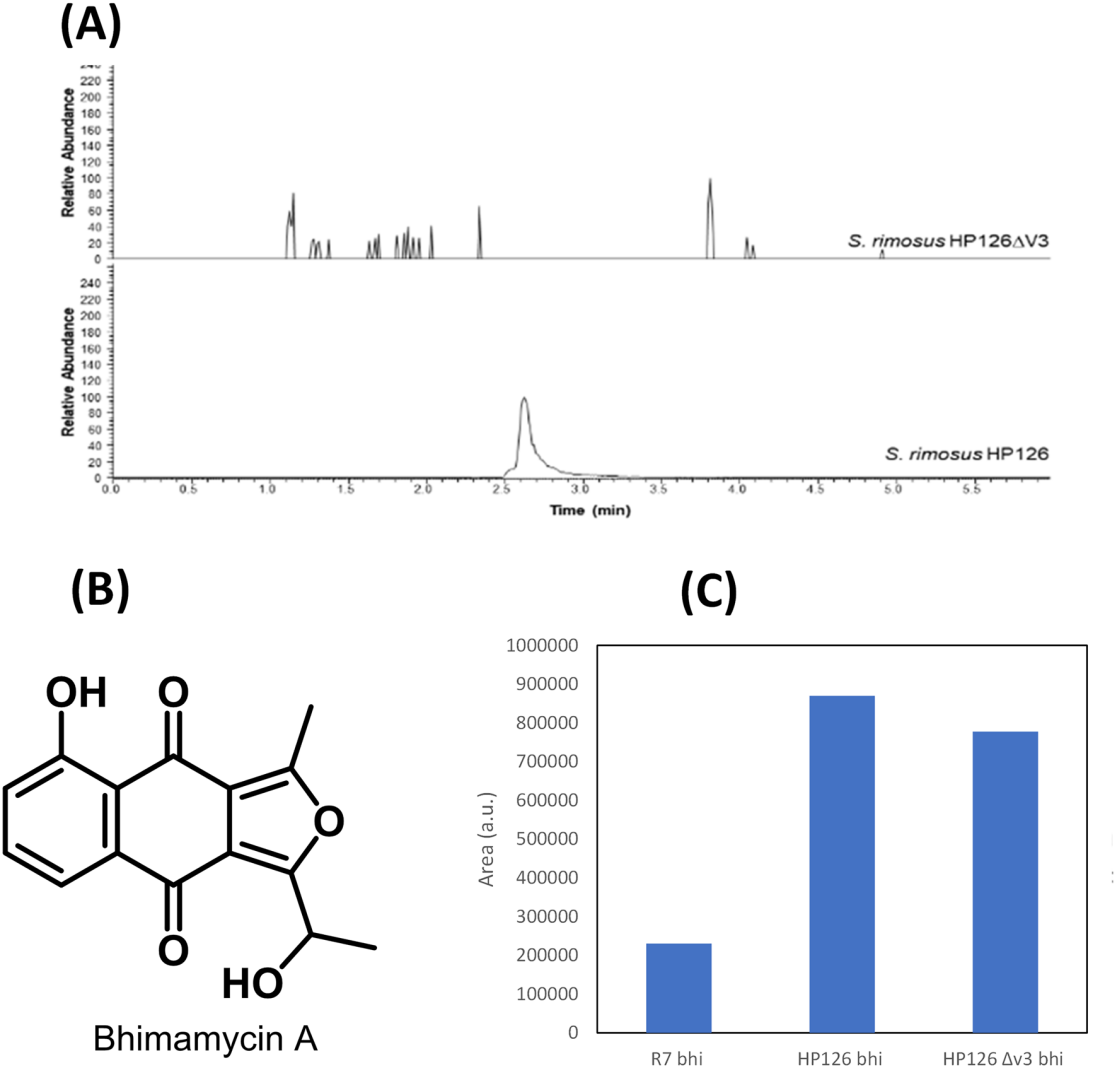
Metabolic engineering of *S. rimosus* for heterologous production of the polyketide bhimamycin. LC‒MS analysis of crude extract from *S. rimosus* HP126 *Δv3* and S. rimosus HP126, showing the extracted ion chromatogram for 461.15–461.20 Da (RT 2.62), which reveals the absence of oxytetracycline in the deletion strain (**A**). Chemical structure of bhimamycin (**B**). Final levels of the product from cultures of *S. rimosus* ATCC 10970, *S. rimosus* HP126, and *S. rimosus Δv3  *(**C**).

Genomic refactoring of *S. rimosus* enables the production of heterologous polyketides. Strain HP126 appeared to be systematically streamlined for oxytetracycline production, showing enhanced expression of the oxytetracycline gene cluster, an increased supply of CoA thioester building blocks (Fig. 6A, B), and the absence of routes to competing byproducts (Fig. 5B). We hypothesized that these acquired traits might be beneficial for synthesizing other polyketides. Therefore, we evaluated *S. rimosus* for heterologous production of bhimamycin A, a polyketide with interesting bioactivity [27]. The coding bhimamycin A gene cluster (*bhi*), containing a type II PKS, was obtained from the *Frankia* sp. CcI3 genome [28] and integrated into the genomes of the wild type and the overproducer. The two obtained mutants were validated by PCR and sequencing. Bhimamycin production was assessed in shake flask cultures. Remarkably, the quantity of heterologous product formed by strain HP126 *bhi* was over three times that of the wild type (Fig. 7B).

Because this mutant still produced substantial amounts of oxytetracycline (data not shown), it was further redesigned. The elimination of its native gene cluster should abolish oxytetracycline formation and thereby enable selective production of bhimamycin. To this end, we constructed *S. rimosus* HP 126 ∆*v3*, a derivative of the classical producer that lacked the entire oxytetracycline biosynthesis-related gene cluster. PCR and sequencing verified the correctness of the desired deletion. As expected, *S. rimosus* HP 126 ∆*v3* did not form any oxytetracycline (Fig. 7A). The mutant grew well on minimal mannitol medium, whereby it showed the same specific growth rate (*µ* = 0.19 h^−1^) during the first phase of cultivation as its parent strain but differed to some extent in growth afterwards. Genome sequencing revealed that the entire oxytetracycline cluster had been removed (Fig. 3A), and the corresponding transcripts were no longer detectable during RNA sequencing (Fig. 3B). However, the cloning was linked to substantial side effects. The plasmid lost the entire sequence that was located right from the ME but simultaneously acquired the right end of the chromosome (including regions 1 and 3). Furthermore, the chromosome and the plasmid contained a few SNPs. The newly duplicated sequence blocks on the plasmid and the chromosome revealed the same SNPs, underlining the constant alignment of the contained sequence information. The genomic rearrangement exhibited a direct impact on gene expression (Fig. 3B). The average expression of genes from duplicated region 3 was significantly enhanced in *S. rimosus* HP 126 ∆*v3*, whereas the other regions did not differ in average expression. This finding revealed that genomic instability and genomic flexibility superimposed the tailored genetic modification, likely causing secondary effects in the obtained mutants.

The transcriptome and proteome appeared relatively similar between the two strains *S. rimosus* HP 126 and HP 126 ∆*v3* (Fig. 5C). A remarkable difference, however, was observed at the metabolomic level. The deletion strain exhibited substantially increased intracellular CoA ester pools, likely caused by the abolished oxytetracycline production (Fig. 6C). In addition, the expression of genes related to CoA metabolism was affected (Table 2). Overall, the chassis strain appeared useful for heterologous production. *S. rimosus* HP 126 ∆*v3 bhi* expressed the bhimamycin A cluster in the genome. Compared to the wild type, the new strain achieved a substantially higher bhimamycin A titer, although it did not fully reach the performance of its parental strain (Fig. 7B). Genetically, the newly obtained strain was almost identical to the parental chassis except for the inserted bhimamycin gene cluster and selected SNPs. Altogether, the novel chassis is a promising platform to selectively derive heterologous polyketides in *S. rimosus.*

## Discussion

The mutagenized strain *S. rimosus* HP126 exhibits a globally streamlined metabolism that drives the production of oxytetracycline. As shown, our work provides a multiomics view of oxytetracycline production in *S. rimosus*. A careful comparison of the genome, transcriptome, proteome, and metabolome between the mutagenized hyperproducer *S. rimosus* HP126 and its ancestor *S. rimosus* ATCC 10970 provided global insight into the changes associated with a 65-fold improvement in oxytetracycline production (Fig. 1).

**Fig. 8:**
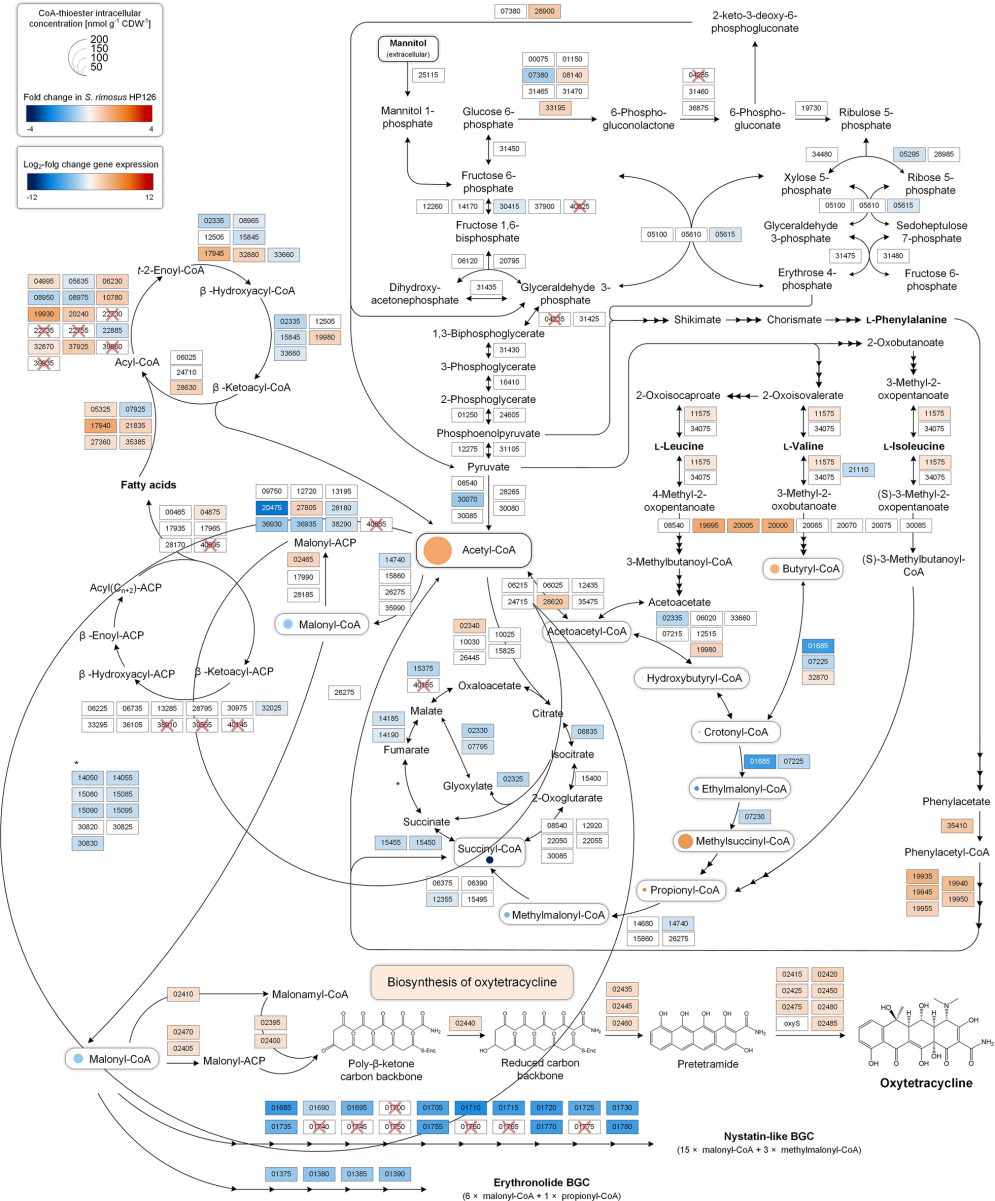
Multiomics profiling of oxytetracycline-producing *S. rimosus*. The data show log_2_-fold changes in the expression of genes encoding enzymes of major catabolic and anabolic pathways and the biosynthesis of oxytetracycline and other malonyl-CoA-derived secondary metabolites in the mutant HP126 (HP) during the production phase (24 h). The expression levels of wild-type *S. rimosus* ATCC 10970 (R7) were used as a reference. Regarding the metabolic pathway repertoire, genome sequencing revealed the deletion and multiplication of different genes. Absent genes in the mutant are marked by a red cross, while a thicker frame around the corresponding rectangle represents an increase in the copy number. The exact copy number for each gene is summarized in Additional file 2. Bubble charts represent the absolute intracellular concentrations of CoA-thioesters and their changes compared to that of *S. rimosus* ATCC 10970. The displayed enzymes were assigned to specific pathways based on KEGG pathway maps and the annotations obtained during DNA and RNA sequencing. n = 3

As shown, the mutagenized hyperproducer acquired systemic novel properties that apparently enabled its superior production performance (Fig. 8). First, the streamlined supply of the oxytetracycline precursors acetyl-CoA and malonyl CoA appeared to be a beneficial trait. Acetyl-CoA and malonyl-CoA are key metabolites that sit at the crossroads between primary carbon metabolism and secondary metabolite production [29]. Notably, the overproducer maintained and even increased the intracellular pools of the two CoA esters to efficiently fuel oxytetracycline biosynthesis, even considering the increased demand (Figs. 6, 8). Interestingly, that multiple pathways of CoA-ester metabolism were affected in transcription, which seemed to systematically boost precursor supply; furthermore, precursor withdrawal into competing pathways was simultaneously diminished (Figs. 6A, 8). This finding suggests that efforts to increase precursor supply increased the formation of oxytetracycline more globally; this was achieved through metabolic engineering strategies for the production of primary bulk chemicals that involve the global redirection of precursor fluxes to increase performance [21, 30, 31, 32, 33]. Second, the oxytetracycline cluster did not carry any single mutation. This finding was surprising, given that overproducers typically express mutated biosynthetic pathway genes encoding enzymes with superior kinetics [34, 35, 36]. Here, the native layout was obviously well balanced [21] to drive oxytetracycline biosynthesis at high flux, and we suggest utilizing its natural sequence. On the other hand, the entire pathway was transcriptionally activated (Figs. 5B, 8), which resulted in significantly more biosynthetic protein (Fig. 5C); thus, further copies of the cluster could be added into the genome [18] or the expression of the cluster could be amplified through stronger promoters [32, 37, 38].

Third, *S. rimosus* HP126 formed loose hyphal mats instead of dense multicellular pellets [39]. Previously, cellular morphology has been identified as a central factor that influences product formation in filamentous microbes [40, 41, 42]. It has been demonstrated that loose mycelial structures enable an improved supply of cultures with oxygen, which is otherwise limited with pellet structures and causes reduced metabolic activities and cell death [41, 43]. Notably, adding talc microparticles to cultures of wild-type *S. rimosus* induced a similar morphology change, as observed here, while simultaneously increasing oxytetracycline production [26]; thus, morphology-related mutations contributed to the improved performance.

Finally, CoA-based biosynthetic routes to competing natural products were substantially downregulated in strain HP126. This contributed to the efficient formation of oxytetracycline through increased precursor availability. Furthermore, oxytetracycline was produced at high selectivity, providing a striking advantage regarding downstream processing. Usually, the process of purifying antibiotics is demanding and expensive and is often complicated by the enormous efforts needed to separate the target product from structurally related natural byproducts [44].

Promising targets for metabolic engineering strategies towards further optimized oxytetracycline production. In strain HP126, almost one-third of the encoded genes exhibited changes in expression; thus, assigning the phenotypic changes to individual mutations was challenging. A few gene candidates, however, showed promise for testing in *S. rimosus* and in other *Streptomyces*. Regarding the engineering of morphology, the reduced expression of cellulose synthase (Table 1) likely contributed to the morphology change in *S. rimosus* HP126, as indicated by the recently demonstrated pellet disintegration effect observed in *matAB-* and *cslA*-deficient strains [45]. Similar effects can be anticipated for the downregulation of chaplin-encoding genes (Additional file 3, Table S1), which were shown to be involved in pellet formation [45].

The fourfold lower concentration of succinyl-CoA in the mutant indicated reduced activity of the TCA cycle, corresponding to the downregulation of multiple genes that encode enzymes in the pathway (Figs. 5A, 8). In turn, oxidative decarboxylation of acetyl-CoA into CO_2_ by enzymes in the TCA cycle was likely reduced in the mutant, and the precursor was saved for oxytetracycline biosynthesis. Actively attenuating the TCA cycle further seems promising [46, 47]. Notably, metabolic engineering of the TCA cycle has improved the production of different acetyl-CoA-based products in other microbes [48].

Furthermore, catabolic pathways should be explored further. The upregulated phenylacetate and branched-chain amino acid degradation pathways seemed to provide extra acetyl-CoA from the degradation of L-phenylalanine, L-valine, and L-isoleucine (Fig. 8), suggesting that these amino acids should be added to the cultures [4, 49]. Ultimately, lipid metabolism emerged as a target for engineering (Fig. 8). The activation of lipases, long chain fatty acyl-CoA synthases, acyl-CoA dehydrogenases, enoyl-CoA hydratases, and thiolases indicated TAG and increased fatty acid degradation in the mutant, providing further supplies of acetyl-CoA and likely stimulating production [50]. This suggests that fatty acyl-CoA synthases are overexpressed to increase the supply of acetyl Co-A and increase the production of oxytetracycline [51]. On the other hand, reduced expression of 3-ketoacyl-ACP synthases indicated that lipid biosynthesis was reduced at the same time. Interestingly, the production of different malonyl-CoA-derived natural products could be increased in *E. coli* due to the downregulation of genes responsible for fatty acid synthesis [52, 53].

*S. rimosus* as a host for heterologous production of secondary metabolites. The mutagenized producer *S. rimosus* HP126 belongs to a family of industrial strains optimized to produce high levels of oxytetracycline [8]. Given its existing genomic traits, re-engineering the mutant to produce other polyketides would be interesting. We hypothesized that introducing a heterologous gene cluster could lead to a bottleneck in oxytetracycline production in strain HP126. Therefore, we removed the core genes of the oxytetracycline gene cluster from the chromosome of *S. rimosus* HP126. *S. rimosus* HP126 *bhi* and *S. rimosus* HP 126 ∆*v3 bhi* could form bhimamycin, underlining the general suitability of the microbe to synthetize heterologous polyketides (Fig. 7). Interestingly, strain HP126 *bhi* accumulated the octaketide more efficiently than HP 126 ∆*v3 bhi*, in contrast to expectations. This finding might indicate that removal of the oxytetracycline cluster or limitations at the pathway regulation level led to imbalances in *S. rimosus* HP 126 ∆*v3 bhi*. Clearly, the reduced efficiency of strain HP 126 ∆*v3 bhi* appeared to be a drawback, as only this design enabled selective production of the heterologous product. *S. rimosus* HP126 *bhi* still formed substantial amounts of oxytetracycline. More work is needed to illustrate this complex picture, including the evaluation of other gene clusters for heterologous expression.

As shown, the mutagenesis process resulted in numerous mutations in strain HP126. The genetic instability of *S. rimosus* resulted in large genomic rearrangements (Fig. 3). In addition to the exchange of large DNA segments between the chromosome and plasmid, deletions, duplications, and even triplications occurred, providing valuable mechanistic insights into genomic plasticity during mutagenesis [54]. The mutations also affected the central conserved part of the chromosome, which is known to contain essential genes in *Streptomyces* [55]. In addition, we observed several hundred single nucleotide changes. Consequently, the mutagenesis-driven mutations and the superimposed natural genetic instability resulted in much more complex changes than those typically observed for other mutagenized bacteria, in which mutations typically remain at the single nucleotide level. This behaviour enabled a larger mutation space, as the large-scale deletion and multiplication events strongly impacted global gene expression (Fig. 3B). In this regard, this behaviour could be an additional driver for strain development. However, the massive chromosomal aberrations that occurred during a single highly precise Red/ET-recombination process, after the oxytetracycline cluster was removed, appear highly undesirable, as the aberrations interfere with the otherwise rational strategy (Fig. 3). Genome stability can vary widely among different strains and species of Streptomyces [56, 57]. It is a complex trait influenced by various factors, including the presence of mobile genetic elements, repetitive sequence motifs, the presence of DNA repair mechanisms, and the environmental conditions in which the bacteria grow [58]. *S. rimosus* has been identified as genetically unstable [59]; as a result, broad rational re-engineering for the heterologous production of natural products might be eventually complicated. In contrast, *S. coelicolor* [60], *S. albus* [28], and *S. lividans* [61] exhibit increased genetic stability, facilitating their use for metabolic engineering.

## Conclusions

To date, mutagenized *Streptomyces* strains have driven the industrial manufacturing of natural products. However, we know little about the molecular traits that enable the multigram-scale production of complex molecules in these mutagenized bacteria, which far exceed the performance of rationally engineered producers. This is also true for oxytetracycline, a decade-lasting antibiotic of success in human and veterinary medicine. To this end, deep multiomics insight into oxytetracycline-producing S. rimosus allowed us to decipher the global nature of the traits that make HP126 a high-efficiency producer. It would be interesting to conduct similar studies on mutagenized producers of other prominent antibiotics, such as erythromycin (*S. erythraeus*)*,* streptomycin (*S. griseus*)*,* and chloramphenicol (*S. venezuela*)*,* and other drugs, such as the antiparasitic agent avermectin (*S. avermitilis*) and the immunosuppressant tacrolimus (FK506) (*S. tsukubaensis*). These studies could reveal general motives that mediate high-level production and be useful for further engineering efforts.

## Materials and methods

### Strains

*S. rimosus* ATCC 10970 (R7) was obtained from the American Type Culture Collection (Manassas, VA, USA). The derivative *S. rimosus* HP126, which belongs to a family of classically derived oxytetracycline hyperproducing strains [18], was kindly donated by Hrvoje Petkovic (University of Ljubljana, Slovenia) within the framework of the MISSION project (031B0611). *E. coli* GB05 (Gene Bridges, Heidelberg, Germany) was used for plasmid DNA manipulation. *E. coli* GB05red (Gene Bridges) was used for λ-red-mediated recombination. *E. coli* ET12567 *pUB307* was used as the helper strain for *E. coli*-*Streptomyces* intergeneric conjugation [62]. All strains and plasmids, including new derivatives that were created during this work, are listed in Table 3. For maintenance, spores (*S. rimosus*) and cells (*E. coli*) were stored in 20% glycerol at −80 °C.

**Table 3:** Strains and plasmids used in this work

Strains	Features	References
***Streptomyces rimosus***		
ATCC 10970	WT (R7)	[9]
ATCC 10970 *bhi*	WT with bhimamycin cluster	This work
HP 126	Oxytetracycline hyperproducing mutant (HP)	[18]
HP 126 *bhi*	HP 126 with bhimamycin cluster	This work
HP 126 ∆*v3*	HP 126 with deleted oxytetracycline cluster (DV3)	This work
HP 126 ∆*v3 bhi*	HP 126 ∆*v3* with bhimamycin cluster	This work
***Escherichia coli***		
GB05-red	Strain used for Red/ET cloning	[89]
GB05	General cloning strain	[89]
ET12567* pUB307*	Donor strain for intergeneric conjugation	[90]
**Plasmids**		
R3A4	Cosmid containing *S. rimosus* chromosome fragment with *otc* cluster	This work
pHSU-OTC3	Derivative of R3A4 with *otc* cluster replaced by *hyg* and *ery* resistance cassettes	This work
patt-sHyg-ery-oriT	Resistance cassette plasmid containing a synthetic fragment with *hyg*, *ery*, *ori*T, *B-CC*, *P-GG,* and *loxP* sites	This work and [65]
pUWLint31	Plasmid containing phiC31 integrase	[65]
1-C9_int_act	Fosmid derivative with a bhimamycin A cluster containing fragment from the chromosome of *Frankia sp*. CcI3	[28]

### Media

Plate cultures were propagated on mannitol soy (MS) agar, which contained 20 g L^−1^ mannitol, 20 g L^−1^ soy flour (Schoenenberger Hensel, Magstadt, Germany), and 20 g L^−1^ agar (Becton & Dickinson, Heidelberg, Germany). For the selection of exconjugants, cells were plated on MS agar that contained nalidixic acid (30 μg mL^−1^) and apramycin (100–2000 μg mL^−1^) or erythromycin (30 μg mL^−1^). Oxytetracycline production in complex medium was conducted in two steps, preculturing and main cultures for production. The vegetative preculture medium (pH 7.0) contained, per litre, 30.0 g of corn starch (Sigma Aldrich, Steinheim, Germany), 5.0 g of NaCl, 4.0 g of CaCO_3_, 4.0 g of corn steep liquor (Sigma Aldrich), 4.0 g of (NH_4_)_2_SO_4_, 3.0 g of soy flour (Schoenenberger Hensel), and 150 mg of KH_2_PO_4_. The oxytetracycline production medium (pH 7.0) contained 75.0 g of corn starch (Sigma Aldrich), 10.0 g of soy flour (Schoenenberger Hensel), 7.0 g of CaCO_3_, 7.0 g of (NH_4_)_2_SO_4_, 2.5 g of corn steep liquor (Sigma Aldrich), 2.0 g of NaCl, 75.0 mg of amylase (Sigma Aldrich), 75 mg KH_2_PO_4_, and 5 mg of CoCl_2_ per litre.

In addition, oxytetracycline production was conducted in minimal sugar medium and involved three steps, as adapted from previous work [4]. The medium for the first preculture contained 20 g of Luria–Bertani (LB) solids (Becton & Dickinson), 10.0 g of sugar (see below), 20.0 g of MOPS, 15.0 g of (NH_4_)_2_SO_4_, 1.0 g of NaCl, 300 mg of KH_2_PO_4_, 55 mg of CaCl_2_, 20 mg of MgSO_4_·H_2_O, 20 mg of FeSO_4_·7H_2_O, 7 mg of CoCl_2_·2H_2_O, 2 mg of FeCl_3_·6H_2_O, 2 mg of MnSO_4_·H_2_O, 1 mg of nicotinamide, 1 mg of riboflavin, 500 µg of pyridoxine·HCl, 500 µg of thiamine·HCl, 500 µg of ZnSO_4_·H_2_O, 200 µg of biotin, 200 µg of CoCl_2_·2H_2_O, 200 µg of Na_2_B_4_O_7_·10H_2_O, 100 µg of 4-aminobenzoic acid, and 100 µg of (NH_4_)_6_Mo_7_O_24_·4H_2_O per litre. For the second preculture, the same medium was used except for the addition of LB. The main culture medium did not contain LB and further exhibited a reduced level of 150 mg L^−1^ of KH_2_PO_4_ [63]. Several alternative sugars were individually tested, including arabinose, xylose, glucose, galactose, fructose, fucose, rhamnose, maltose, and mannitol. The production of bhimamycin included a preculture step in TSB medium (30 g L^−1^, Ooid, Basingstoke, United Kingdom). The main cultures for production were conducted in DNPM medium [64].

### Molecular biology and genetic engineering

Genetic engineering of *S. rimosus* was performed following previous protocols [65]. For the isolation of genomic DNA, strains were grown on TSB medium (Ooid, Basingstoke, United Kingdom). For the selection of exconjugants, cells were plated on MS agar containing nalidixic acid (30 μg mL^−1^) and apramycin (100–2000 μg mL^−1^) or erythromycin (30 μg mL^−1^). During cloning, *E. coli* strains were grown in LB medium (20 g L^−1^, Becton & Dickinson) containing apramycin (100 μg mL^−1^), kanamycin (50 μg mL^−1^), chloramphenicol (12.5 μg mL^−1^), and hygromycin (120 μg mL^−1^) when necessary. All primers used are listed in Additional file 1: Table S1.

For deletion of the oxytetracycline cluster, a cosmid library of *S. rimosus* HP126 was prepared in the pCos15A *gusA* vector using the EpiCentre CopyControl Fosmid Library Production Kit (Lucigen, Middleton, WI, USA). In short, a library of 30 to 40 kb genomic DNA fragments was constructed according to the manufacturer’s protocol using genomic DNA of *S. rimosus* HP126 that was partially digested with MssI. The purified fragments were ligated into the linearized pCos15A *gusA* vector. Then, the ligated constructs were packed into λ phage for *E. coli* EPI300 infection. The packaged library was plated on LB agar plates containing 12.5 µg mL^−1^ chloramphenicol and grown overnight at 37 °C. Approximately 1,500 single colonies were picked and inoculated into individual wells of 96-well plates filled with 20% glycerol. The arrayed cosmid library was stored at − 80 °C. The cosmid clone R3A4 (37 kb) was identified to contain a 29 kb fragment that comprised the entire oxytetracycline gene cluster plus upstream and downstream flanking regions. The oxytetracycline cluster was deleted from the genome of *S. rimosus* HP126 using an iterative marker excision system [65]. The cosmid clone R3A4 was used to construct the deletion plasmid pHSU-OTC3 (see above). The antibiotic cassettes for hygromycin and erythromycin together with OriT were flanked by a P-GG and a B-CC site for ΦC31 integrase and amplified by PCR with the primers HSU-OTC35 and HSU-OTC34 using the patt-sHyg-ery-oriT plasmid as the template. Utilizing PCR-based λ-red recombination, the amplified hyg-ery cassette was then used to delete 23 kb out of 29 kb of the oxytetracycline gene cluster on the cosmid R3A4, The entire sequence from gene *oxyA* to gene to *otcA* was removed and the deletion construct pHSU-OTC3 was generated. The resulting cosmid was subsequently introduced into *S. rimosus* HP126 by conjugation. Single crossover mutants were selected via erythromycin resistance. Double crossover mutants were then screened on MS agar supplemented with erythromycin and 70 µg mL^−1^ X-Gluc for blue‒white selection [66]. Finally, the resistance markers were excised from the chromosome by expressing the ΦC31 integrase from pUWLint31 [65]. The deletion of the oxytetracycline cluster was confirmed by PCR using the primers HSU-OTC41 and HSU-OTC46, and the obtained PCR fragments were sequenced. The resulting strain was designated *S. rimosus CLEAN*.

For heterologous bhimamycin A production, the cryptic type II PKS for bhimamycin A biosynthesis was captured from the *Frankia* sp. CcI3 genome and expressed in *S. rimosus* ATCC 10970, *S. rimosus* HP126, and *S. rimosus* HP 126 ∆*v3* as described previously [28].

### Oxytetracycline production in complex medium

Strains were propagated on MS agar at 30 °C for 7 days, and the sporulating mycelium was amended with 5 mL sterile deionized water. Afterwards, spores were collected using a glass cell spreader and the obtained suspension was centrifuged (5500 × *g*, 4 °C, 6 min). The obtained pellet was resuspended in 4 mL of 20% ice-cold glycerol. The content of viable spores in the suspension was determined using serial dilutions that were prepared in triplicate on tryptone soy broth agar (30 g L^−1^, Sigma Aldrich). The spore suspension was used to inoculate a preculture in vegetative medium (50 mL in 500 mL non-baffled shake flasks) to an initial concentration of 1 × 10^7^ spores mL^−1^. The preculture was incubated for 24 h on a rotary shaker at 30 °C, 230 rpm, and 80% humidity (Multitron, Infors AG, Bottmingen, Switzerland). Afterwards, 5 mL of preculture broth was used to inoculate the main culture in production medium, which was then incubated under the same conditions. Experiments were conducted in triplicate.

### Production of oxytetracycline in minimal medium

A first preculture was inoculated to an initial spore concentration of 1 × 10^7^ spores mL^−1^ and grown for 24 h. Subsequently, the cells were harvested (8,500 × *g*, 20 °C, 5 min), washed once, and used to inoculate the second preculture to an initial cell dry mass (CDM) concentration of 0.50 g L^−1^. The second preculture was grown for 15 h, harvested, washed (8,500 × *g*, 20 °C, 7 min), and inoculated into the main culture to an initial CDM concentration of 0.50 g L^−1^. All cultures were incubated in 500 mL baffled shake flasks filled with 50 mL medium on a rotary shaker at 30 °C, 230 rpm and 80% relative humidity. For initial substrate screening tests, the main cultures were incubated in the same incubator but in reagent glass tubes (22 mL) filled with 5 mL of medium (30 °C, 230 rpm, 80% humidity). Experiments were carried out in triplicate.

### Heterologous production of bhimamycin A

Recombinant clones of *S. rimosus* ATCC 10970, *S. rimosus* HP126, and *S. rimosus* HP126 ∆*v3,* which each carried the heterologous PKS gene cluster for the biosynthesis of bhimamycin A, were cultivated in two steps. First, the strains were grown in 15 mL TSB medium (150 mL baffled shake flasks) for 48 h. Afterwards, 1 mL of preculture broth was used to inoculate 50 mL of DNPM medium, which was filled in 150 mL baffled shake flasks. All strains were cultivated at 30 °C, 230 rpm, and 80% humidity. Experiments were performed in triplicate.

### Quantification of cell concentration

Cells were collected from broth by vacuum filtration using a nitrocellulose filter (0.2 µm, Sartorius, Göttingen, Germany) and washed twice with 0.9% NaCl. Subsequently, the CDM on the filter was gravimetrically measured (HB43-S, Mettler-Toledo, Columbus, OH, USA) [3]. In addition, the cell concentration was analysed as the optical density (OD_660_) at a wavelength of 600 nm. For the early phase of growth, a linear correlation between CDM and OD_660_ was obtained, as previously observed for other strains of *Streptomyces* [3]. Notably, the correlation factor differed between the strains as follows: CDM [g L^−1^] = 0.917 × OD_600_ (*S. rimosus* ATCC 10970) and CDM [g L^−1^] = 0.412 × OD_600_ (*S. rimosus* HP126 and *S. rimosus* HP126 ∆*v3*). Through these correlations, we could infer the CDM from OD_660_ readings during the first 8 h of cultivation, when samples did not contain enough cells for precise gravimetric analysis [3]. Measurements were conducted in triplicate.

### Quantification of mannitol

Mannitol was quantified in culture supernatant by HPLC (Agilent 1260 Infinity Series, Agilent Technologies, Waldbronn, Germany) using ion-moderated partition chromatography on a reversed-phase column as the stationary phase (Aminex HPX-87H, 300 × 7.8 mm, 9 µm, Bio-Rad, Hercules, CA, USA) and 7 mM H_2_SO_4_ (0.5 mL min^−1^, 65 °C) as the mobile phase [37]. Mannitol was detected using the refractive index, and quantification was based on external standards.

### Quantification of phosphate

Phosphate was analysed by ion chromatography (Integrion IC, Thermo Scientific, Karlsruhe, Germany) using a carbonate-selective anion-exchange column (Dionex IonPac AS9-HC, 250 × 2 mm, 9 µm, Thermo Scientific) as the stationary phase and 12 mM Na_2_CO_3_ (0.25 mL min^−1^, 30 °C) as the mobile phase [49]. Quantification was based on suppressed conductivity detection and external standards.

### Quantification of oxytetracycline

For sample pretreatment, 250 µL of broth was acidified to pH 1.5 using 37% HCl, followed by 15 min of incubation on ice and centrifugation (16,000 × *g*, 4 °C, 10 min). The obtained supernatant was analysed by HPLC (Agilent 1260 Infinity Series, Agilent Technologies). The system was operated with a reversed-phase column (Nucleodur C18 Isis, 100 × 3 mm, 3 µm, Macherey–Nagel, Düren, Germany) and a gradient of 0.1% formic acid (eluent A) and methanol (eluent B) at 0.8 mL min^−1^ and 40 °C: 0–7 min, 0% B; 7–8 min, 0–32% B; 8–11 min, 32–100% B; 11–15 min, 100–0% B. Oxytetracycline was detected at 275 nm using a diode-array detector and quantified using external standards.

### Quantification of bhimamycin A

Bhimamycin A was quantified using LC‒MS as described previously [28]. In short, the analytes of interest were extracted from culture broth using ethyl acetate, followed by evaporation of the solvent and dissolution of the crude extract in methanol. LC‒MS analysis was then conducted on a high-resolution ESI QTOF (Bruker Maxis II, Bruker Daltonics, Billerica, MA, USA) coupled to an LC (Thermo Dionex Ultimate 3000 RS, Thermo Fisher). Separation was based on a reversed-phase column (ACQUITY BEH C18, 100 × 2.1 mm, 1.7 µm, Waters, Eschborn, Germany) at a flow rate of 0.6 mL min^−1^ using a linear gradient of eluent A (0.1% formic acid in water) and eluent B (0.1% formic acid in acetonitrile) with an increase in eluent B from 0 to 95% over 18 min. Relative quantification was performed based on the obtained peak area.

### Microscopy of cell aggregates

Ten microlitres of culture sample was transferred to a glass slide, covered with a coverslip, and analysed by bright-field microscopy (Olympus IX, Hamburg, Germany). ImageJ 1.53 software was used to automatically process microscopic pictures of the cultures [40]. For morphological characterization, the maximum Feret diameter of *S. rimosus* pellets was determined. The value represents the smallest circle into which a pellet could fit [26]. At least 100 aggregates were analysed per sample.

### Genome analysis

Genomic DNAs of strains R7, HP126, and ∆v3 were isolated via a NucleoSpin Microbial DNA kit (MACHEREY–NAGEL). Long and short DNA reads were generated by Nanopore and Illumina sequencing, respectively. For library preparation, the TruSeq DNA PCR-free high-throughput library prep kit (Illumina) and the SQK-LSK109 sequencing kit with barcode extension EXP-NBD104 (Oxford Nanopore Technologies) were used without prior shearing of the DNA. To generate the short reads, a 2 × 300-nucleotide run (MiSeq reagent kit v3, 600 cycles) was executed. The long reads were generated on a GridION platform using an R9.41 flow cell. Base calling and demultiplexing for the ONT data were performed using GUPPY v4.0.1 with the high accuracy base calling model. The assemblies were performed using flye v.2.9 [67] and canu v2.1 [68] for the Nanopore long read data and newbler v2.8 [69] for the Illumina short read data. After the flye- and canu-based assemblies were polished using medaka v1.5.0 and pilon v1.22 [70] and Bowtie2 [71] for mapping, all assemblies were combined and manually finished in consed v28.0 [72], resulting in one contig per replicon. In all three strains, one contig represented the linear genome, and the other contig represented the linear plasmid. All genomes were annotated using the prokka v1.11 pipeline [73]. The functional annotation of the genes, designated by SRIMR7 numbers on basis of the sequenced genomes, is given in Additional file 2. Genomic rearrangements were determined using BLASTN by comparing the genomes against each other, using 99% sequence identity as a filter criterion.

### Transcriptome analysis

Transcription profiling was performed as described previously [74]. In short, cells were collected by centrifugation (20,000×*g*, 4 °C, 1 min), and the obtained pellet was immediately frozen in liquid nitrogen. Total RNA was isolated (Quick-RNA Miniprep Plus kit, Zymo Research, Freiburg, Germany), purified (RNA Clean & Concentrator-5 kit, Zymo Research, Freiburg, Germany), and quantified (DropSense 16, Trine an NV, Gentbrugge, Belgium). The quality of the obtained RNA was validated (RNA 6000 Nano kit, Agilent 2100 Bioanalyzer, Agilent Technologies). Then, 2.5 μg of total RNA (RIN > 9) was depleted from rRNA (Ribo-Zero rRNA Removal Kit Bacteria, Illumina, San Diego, CA, USA). The completeness of the depletion was checked (Agilent RNA Pico 6000 kit, Agilent 2100 Bioanalyzer, Agilent Technologies). The obtained mRNA was converted to a cDNA library (TruSeq Stranded mRNA Sample Preparation Guide, Illumina, San Diego, CA, USA). After quality control and quantification (Agilent High Sensitivity DNA kit, Agilent 2100 Bioanalyzer, Agilent Technologies), all created cDNA libraries were sequenced using a 75-base read length (NextSeq 500, Illumina). The obtained reads were mapped to the genome of strain R7 (chromosome and plasmid) with Bowtie2 using standard settings [71] except for increasing the maximal allowed distance for paired reads to 600 bases. ReadXplorer 2.2.3 was used to visualize read alignments [75]. For counting reads mapping to gene features, FeatureCounts v.2.0.0 [76] was applied using the parameters -M -O and -s 1. With the resulting data, DESeq2 [77] was used to QC the datasets via several methods, including calculation of the sample-to-sample distances (Additional file 1, Fig. S2) and PCA (Additional file 1, Fig. S3). In addition, DESeq2 was used to calculate DGE datasets. Raw datasets (sequenced reads) as well as processed datasets (input matrix and normalized read counts from DESeq2) are available from GEO (GSE168592). For statistical analysis, Student's t test was carried out, and the data were filtered for genes with a log2‐fold change ≥ 1 (p ≤ 0.05). Data analysis and visualization were conducted using the software gplots [78, 79].

### Proteome analysis

For protein isolation and processing, a single-tube preparation protocol was applied [80]. Bacterial cells were resuspended in 200 µl ammonium bicarbonate (100 mM). The suspension was transferred into a tube containing glass beads, and cells were disrupted in three cycles for 20 s with cooling breaks in between. Then, 100 µl of trifluoroethanol and 5 µl of 200 mM dithiothreitol were mixed with 100 µl of the obtained cell lysate and incubated for 60 min at 60 °C. The cysteine residues were alkylated by the addition of 20 µl of iodoacetamide (200 mM) for 90 min at RT in the dark. The reaction was stopped by adding 5 µl of 200 mM dithiothreitol, followed by incubation at RT for 60 min. For tryptic digestion, the samples were diluted 1:10 with ammonium bicarbonate (100 mM) and incubated at 37 °C overnight (Trypsin Gold, Promega). Digested peptides were purified using SepPak columns (Waters, Milford, United States). Peptide quantification was performed using a Nanodrop 2000 (Thermo Fisher Scientific, Germany).

The peptides were analysed via LC‒MS/MS using a nano-LC (Ultimate 3000, Thermo Fisher Scientific, Germany) coupled to an ESI-Orbitrap MS/MS (QExactive Plus, Thermo Fisher Scientific, Germany). The gradient length of the Acclaim PepMap C18 2UM 75UMx250MM (Thermo Fisher Scientific, Dreieich, Germany) analytical column was adjusted to 67 min from 4 to 50% of 80% ACN and 0.08% FA at a flow rate of 300 nL min^−1^. ESI-Orbitrap-MS measurements were carried out in data-dependent top 10 acquisition mode. All samples were measured in full MS mode using a resolution of 70.000 (AGC target of 3e6 and 64 ms maximum IT). For dd-MS2, a resolution of 17.500, an AGC target of 2e5, and a maximum IT of 100 ms were used.

The database search was performed with MaxQuant version 1.6.14.0 [81]. For the variable modifications, methionine oxidation and N-terminal acetylation were set. Fixed modifications were set for the carbamido-methylation of cysteine. For digestion, trypsin was selected with a maximum of 2 missed cleavages. Label-free quantification was set to LFQ, and fast LFQ was enabled. The database used for identification was built from the coding sequences of CP094298.1 (R7 chromosome) and CP094299.1 (R7 plasmid), which together contained 8,365 entries. Only unique peptides were used for quantification. For the FDR calculation, the decoy mode was set to random. The remaining settings were set to default.

The statistical analysis of the database search with MaxQuant was performed in Perseus 1.6.14.0 [82]. The data were transformed using the log2(x) equation. Proteins that were found by MaxQuant as “Only identified by side”, “Reverse” and “Potential contamination” were filtered out. In addition, the proteins with less than two unique peptides were filtered out, as well as proteins without at least 3 valid values in total. The imputation of missing values was performed with a width of 0.3 and a downshift of 1.8 for each column separately.

### Quantification of intracellular CoA-thioesters

The analysis of CoA-thioesters was adapted from previous work [83]. In short, culture samples (containing 5 mg CDM) were quenched with extraction buffer (25 mM formic acid, 95% acetonitrile, −20 °C), extracted through a 10 min series of mixing and cooling steps on ice, followed by centrifugation (15,000×*g*, 4 °C, 10 min), and collection of the obtained supernatant in 10 mL supercold deionized water. The remaining cell pellet was washed twice with 8 mL of supercold water, and all supernatants were combined afterwards. The extract was frozen in liquid nitrogen, freeze-dried, and subsequently resuspended in 500 µL buffer (25 mM ammonium formate, 2% MeOH, pH 3.0, 4 °C). The buffered extract was clarified from debris (15,000 × *g*, 4 °C, 10 min). CoA thioester quantification in the extract was carried out using triple quadrupole MS (QTRAP 6500, AB Sciex, Darmstadt, Germany) coupled to an LC system (Agilent 1290 Infinity System, Waldbronn, Germany). The analytes of interest were separated on a reversed-phase column (Gemini C18, 100 × 2.1 mm, 1.5 µm, Phenomenex, Aschaffenburg, Germany) at 40 °C and a flow rate of 0.6 mL min^−1^ using a gradient of eluent A (50 mM formic acid, pH 8.1) and eluent B (methanol). The fraction of eluent B was as follows: 0–12 min, 0–15%; 12–16 min, 15–100%; 16–18 min, 100%; 18–20 min, 100–0%; and 20–25 min, 0%. For absolute quantification, a ^13^C-labelled cell extract was used as an internal standard [4]. For this purpose, *S. rimosus* was grown on 99% [^13^C_6_] mannitol (Omicron, Biochemicals, South Bend, IN, United States). To obtain a fully ^13^C-enriched cell extract, the second preculture was grown on 99% [^13^C_6_] mannitol as well, and the inoculum size for the second preculture and the main culture remained below 1% of the final harvested cell amount [84]. The extraction was conducted as described above. The concentration of the individual ^13^C-CoA thioesters in the extract was determined using synthetic standards. For later absolute quantification in growth experiments, an appropriate amount of ^13^C extract was added to culture samples during the initial quenching step [83].

### Supplementary Information

**Additional file 1: Fig. S1.** Impact of the nutrient environment on oxytetracycline production in *S. rimosus*. Production by strains ATCC 10970 and HP126 in complex starch-based medium (A) and production by strain ATCC 10970 in minimal medium with different sugars as carbon sources (10 g L^−1^) (B). The data represent the final oxytetracycline titer at the end of cultivation after 120 h. n = 3. The complex starch-based medium did not appear to be optimal for the planned systems biology analyses. It could not be ruled out that the two strains use the contained proportions of soybean meal and corn steep liquor differently, which would have significantly complicated the interpretation of the multiomics data [4]. Therefore, a minimal, fully defined medium was desired for the subsequent investigations, which could reveal the phenotypic differences between the strains [23]. A mineral medium containing a limiting amount of phosphate to trigger the formation of oxytetracycline [63] was tested for this purpose. The basic salt mixture was amended with the following different sugars as the sole carbon and energy source to identify the most suitable setup: five hexoses (galactose, glucose, fructose, fucose, rhamnose), two pentoses (arabinose, xylose), a sugar alcohol (mannitol), and a disaccharide (maltose). Test tubes filled with 1 mL of medium were used to screen for the substrate that led to the best production in the wild type (B). Notably, the strain produced measurable amounts of oxytetracycline on all tested carbon sources, revealing a broad substrate spectrum. The highest titer (17 mg L^−1^) was observed on mannitol. It was almost twice as high as that achieved on the hexose sugars. In comparison, oxytetracycline formation on the two pentoses and maltose was even smaller (between 1 and 2 mg L^−1^), indicating that the carbon source substantially impacted production efficiency. Based on the outcome, mannitol appeared most suitable. A test tube culture with strain HP126 on mannitol-based medium yielded 200 mg L^−1^ oxytetracycline, almost tenfold more than that observed for the wild type. Obviously, the minimal medium with sugar alcohol reflected the different production performance of both strains very well, as desired for the planned systems biology comparison. This medium was therefore selected for further studies. Generally, all test tube and shake flask cultures revealed excellent reproducibility (Fig. 2). **Fig. S2.** Heatmap of the sample-to-sample distances. The read count data were fed to DESeq2 [77] to calculate normalized read counts. After regularized log transformation was performed with blind dispersion estimation, the sample-to-sample distances were calculated and used for hierarchical clustering, which was then visualized using pheatmap [86]. Samples were taken from cultures of *S. rimosus* ATCC 10970 (R7), its mutagenized oxytetracycline-overproducing derivative HP126 (HP) and the chassis strain HP126 ∆v3 (DV3) after 24 h. n = 3. **Fig. S3.** Statistical evaluation of gene expression profiles using PCA. For the calculation of normalized read counts, the raw read count data were processed by DESeq2 [87], including regularized log transformation (with blind dispersion estimation enabled). Subsequently, PCA was performed and visualized using ggplot2 [88]. Samples were taken from cultures of *S. rimosus* ATCC 10970 (R7), its mutagenized oxytetracycline-overproducing derivative HP126 (HP) and the chassis strain HP126 ∆v3 (DV3) after 24 h. n = 3. **Table S1.** Copy number of the genes related to major catabolic and anabolic processes in *S. rimosus* ATCC10970, *S. rimosus* HP126 and *S. rimosus* HP126 Δv3. **Table S2.** BlastP identification of morphology regulators and their expression levels in *S. rimosus* HP126 and *S. rimosus* HP126 Δv3.

**Additional file 2.** Functional annotation of genes in *S. rimosus*, designated by SRIMR7 numbers on basis of the sequenced genomes.

**Additional file 3.** Gene expression data on cell synthesis and morphology, oxytetracycline biosynthesis, lipid and fatty acid metabolism, and biosynthesis of other secondary metabolites.

## Data Availability

All DNA data (raw reads and annotated genomes) are available via BioProjects PRJNA756560 (R7), PRJNA853543 (HP126), and PRJNA853544 (∆v3). Transcriptome data are accessible via GEO GSE232318. The mass spectrometry proteomics data have been deposited in the ProteomeXchange Consortium via the PRIDE [85] partner repository with the dataset identifier PXD042182.

## Availability of data and materials

The dataset(s) supporting the conclusions of this article are all included within the article.
