# Supplementary material for: Systems biology of industrial oxytetracycline production in Streptomyces rimosus: the secrets of a mutagenized hyperproducer
Source: Microb Cell Fact. 2023 Oct 28;22:222. doi: 10.1186/s12934-023-02215-x (PMC10612213; doi:10.1186/s12934-023-02215-x)
Supplement: Supplementary file 1 — Additional file 1: Fig. S1. Impact of the nutrient environment on oxytetracycline production in S. rimosus. Production by strains ATCC 10970 and HP126 in complex starch-based medium (A) and production by strain ATCC 10970 in minimal medium with different sugars as carbon sources (10 g L−1) (B). The data represent the final oxytetracycline titer at the end of cultivation after 120 h. n = 3. The complex starch-based medium did not appear to be optimal for the planned systems biology analyses. It could not be ruled out that the two strains use the contained proportions of soybean meal and corn steep liquor differently, which would have significantly complicated the interpretation of the multiomics data [4]. Therefore, a minimal, fully defined medium was desired for the subsequent investigations, which could reveal the phenotypic differences between the strains [23]. A mineral medium containing a limiting amount of phosphate to trigger the formation of oxytetracycline [63] was tested for this purpose. The basic salt mixture was amended with the following different sugars as the sole carbon and energy source to identify the most suitable setup: five hexoses (galactose, glucose, fructose, fucose, rhamnose), two pentoses (arabinose, xylose), a sugar alcohol (mannitol), and a disaccharide (maltose). Test tubes filled with 1 mL of medium were used to screen for the substrate that led to the best production in the wild type (B). Notably, the strain produced measurable amounts of oxytetracycline on all tested carbon sources, revealing a broad substrate spectrum. The highest titer (17 mg L−1) was observed on mannitol. It was almost twice as high as that achieved on the hexose sugars. In comparison, oxytetracycline formation on the two pentoses and maltose was even smaller (between 1 and 2 mg L−1), indicating that the carbon source substantially impacted production efficiency. Based on the outcome, mannitol appeared most suitable. A test tube culture with strain HP126 on mannito [file 12934_2023_2215_MOESM1_ESM.pdf]

Additional file 1 to

Systems biology of industrial oxytetracycline production in *Streptomyces rimosus*: the secrets of a mutagenized hyperproducer

Microbial Cell Factories

Selma Beganovic[1], Christian Rückert-Reed[2], Hilda Sucipto[3], Wei Shu[1], Lars Gläser[1], Thomas Patschkowski[2], Ben Struck[2], Jörn Kalinowski[2], Andriy Luzhetskyy[3], and Christoph Wittmann[1*]

[1] Institute of Systems Biotechnology, Saarland University, Saarbrücken, Germany

[2] Centre for Biotechnology, Bielefeld University, Bielefeld, Germany

[3] Department of Pharmacy, Saarland University, Saarbrücken, Germany

* Campus A1 5, 66123 Saarbrücken, Germany, christoph.wittmann@uni-saarland.de, Phone: +49-681-302-71970, FAX: -71972

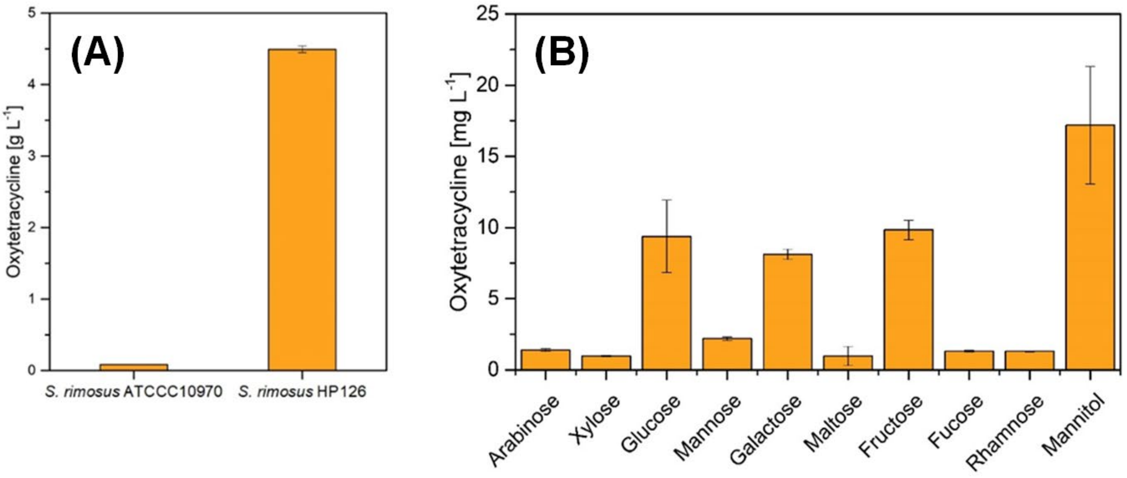

Figure S1. Impact of the nutrient environment on oxytetracycline production in strains of *S. rimosus*. Production by strains ATCC 10970 and HP126 in complex starch-based medium (A). Production by strain ATCC 10970 in minimal medium with different sugars as carbon source (10 g L^{-1}). The data represent the final oxytetracycline titer at the end of cultivation after 120 h. n=3. The complex starch-based medium did not appear to be optimal for the planned systems biology analyses (A). It could not be ruled out that the two strains would use the contained proportions of soybean meal and corn steep liquor differently, which would have significantly complicated the interpretation of the multi-omics data (Gläser, et al., 2021). Therefore, a minimal, fully defined medium was desired for the subsequent investigations, which, additionally, could visualize the phenotypic differences between the strains (Kohlstedt, et al., 2014). A mineral medium, containing a limiting amount of phosphate to trigger the formation of oxytetracycline (McDowall, et al., 1999), was tested for this purpose. The basic salt mixture was amended with nine different sugars as the sole carbon and energy source to identify the most suitable set-up: five hexoses (galactose, glucose, fructose, fucose, rhamnose), two pentoses (arabinose, xylose), a sugar alcohol (mannitol), and a disaccharide (maltose). Test tubes, filled with 1 mL medium, were used to screen for the substrate that enabled best production in the wild type (B). Notably, the strain produced measurable amounts of oxytetracycline on all tested carbon sources, revealing a

broad substrate spectrum. The highest titer (17 mg L^{-1}) was observed on mannitol. It was almost twice as high than that achieved on the hexose sugars. In comparison, oxytetracycline formation on the two pentoses and maltose was even smaller (between 1 and 2 mg L^{-1}), indicating a substantial impact of the carbon source on production efficiency. Based on the outcome, mannitol appeared most suitable. A test tube culture with strain HP126 on mannitol-based medium yielded 200 mg L^{-1} oxytetracycline, almost ten-old more than observed for the wild type. Obviously, the minimal medium with the sugar alcohol reflected the different production performance of both strains very well, as desired for the planned systems biology comparison. This medium was therefore selected for the further studies. Generally, all test tube and shake flask cultures revealed excellent reproducibility (**Fig. 2**).

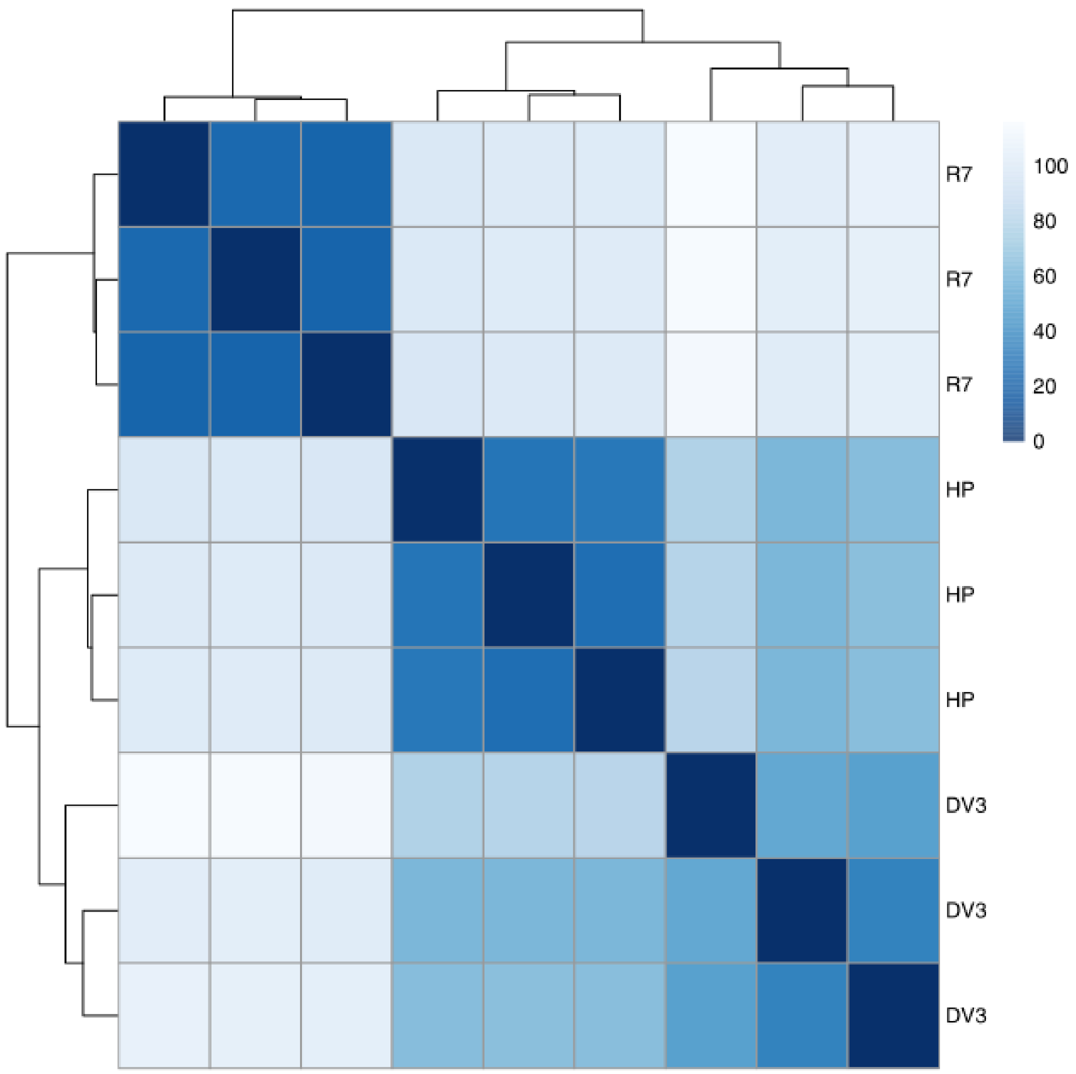

Fig. S2: Heatmap of the sample-to-sample distances. The read count data were fed to DESeq2 (Love, et al., 2014) to calculate normalized read counts. After regularized log transformation with blind dispersion estimation enabled, the sample to sample distances were calculated and used for hierarchical clustering, which in turn was visualized using pheatmap (Kolde, 2019). Samples were taken from cultures of *S. rimosus* ATCC 10970 (R7), its mutagenized oxytetracycline-overproducing derivative HP126 (HP) and the chassis strain HP126 Dv3 (DV3) after 24 h. n=3.

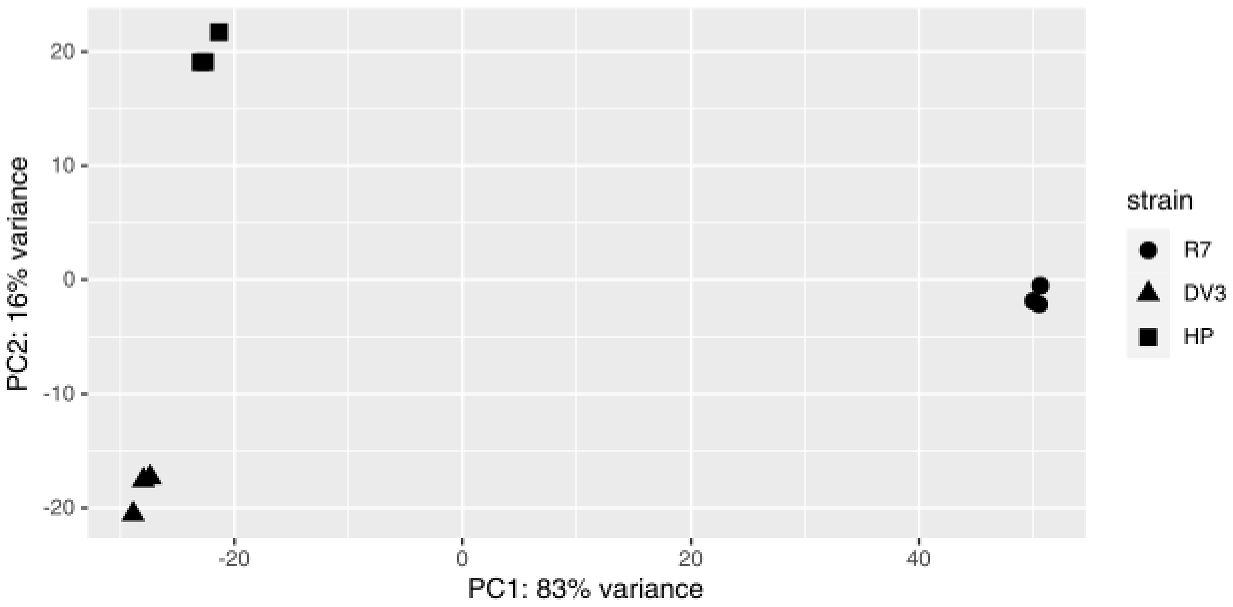

Fig. S3: Statistical evaluation of gene expression profiles using PCA. For the calculation of normalized read counts, the raw read count data were processed by DESeq2 (Love, et al., 2014), including regularized log transformation (with blind dispersion estimation enabled). Subsequently, PCA was performed and visualized using ggplot2 (Wickham, et al., 2016). Samples were taken from cultures of *S. rimosus* ATCC 10970 (R7), its mutagenized oxytetracycline-overproducing derivative HP126 (HP) and the chassis strain HP126 Dv3 (DV3) after 24 h. n=3.

Table S1. Copy number of the genes related to major catabolic and anabolic processes in *S. rimosus* ATCC10970, *S. rimosus* HP126, and *S. rimosus* HP126 Δv3.

Locus tag	*S. rimosus* ATCC10970	*S. rimosus* HP126	*S. rimosus* HP126 Δv3	Locus tag	*S. rimosus* ATCC10970	*S. rimosus* HP126	*S. rimosus* HP126 Δv3
SRIMR7_00075	1	3	3	SRIMR7_15495	1	1	1
SRIMR7_00465	1	3	3	SRIMR7_15825	1	1	1
SRIMR7_01150	1	2	3	SRIMR7_15845	1	1	1
SRIMR7_01250	1	2	3	SRIMR7_15860	1	1	1
SRIMR7_01375	1	2	3	SRIMR7_16410	1	1	1
SRIMR7_01380	1	2	3	SRIMR7_17940	1	1	1
SRIMR7_01385	1	2	3	SRIMR7_17945	1	1	1
SRIMR7_01390	1	2	3	SRIMR7_17965	1	1	1
SRIMR7_01685	1	2	3	SRIMR7_17990	1	1	1
SRIMR7_01690	5	6	7	SRIMR7_19730	1	1	1
SRIMR7_01695	1	2	3	SRIMR7_19930	1	1	1
SRIMR7_01705	1	2	3	SRIMR7_19935	1	1	1
SRIMR7_01710	1	2	3	SRIMR7_19940	1	1	1
SRIMR7_01715	1	2	3	SRIMR7_19945	1	1	1
SRIMR7_01720	1	2	3	SRIMR7_19950	1	1	1
SRIMR7_01725	1	2	3	SRIMR7_19955	1	1	1
SRIMR7_01730	1	2	3	SRIMR7_19980	1	1	1
SRIMR7_01735	1	2	3	SRIMR7_19995	1	1	1
SRIMR7_01755	1	2	3	SRIMR7_20000	1	1	1
SRIMR7_01770	1	2	3	SRIMR7_20005	1	1	1
SRIMR7_01780	1	2	3	SRIMR7_20065	1	1	1
SRIMR7_02325	1	1	1	SRIMR7_20070	1	1	1
SRIMR7_02330	1	1	1	SRIMR7_20075	1	1	1
SRIMR7_02335	1	1	1	SRIMR7_20240	1	1	1
SRIMR7_02340	1	1	1	SRIMR7_20475	1	1	1
SRIMR7_02465	1	1	0	SRIMR7_20795	1	1	1
SRIMR7_04875	1	1	2	SRIMR7_21110	1	1	1
SRIMR7_04995	1	1	2	SRIMR7_21835	1	1	1
SRIMR7_05100	1	1	2	SRIMR7_22050	1	1	1
SRIMR7_05295	1	1	2	SRIMR7_22055	1	1	1
SRIMR7_05325	1	1	2	SRIMR7_22885	1	1	1
SRIMR7_05610	1	1	2	SRIMR7_24605	1	1	1

SRIMR7_05615	1	1	2	SRIMR7_24710	1	1	1
SRIMR7_05635	1	1	2	SRIMR7_24715	1	1	1
SRIMR7_06020	1	1	2	SRIMR7_25115	1	1	1
SRIMR7_06025	1	1	2	SRIMR7_26275	1	1	1
SRIMR7_06025	1	1	2	SRIMR7_26445	1	1	1
SRIMR7_06120	1	1	2	SRIMR7_26985	1	1	1
SRIMR7_06215	1	1	2	SRIMR7_27360	1	1	1
SRIMR7_06225	1	1	2	SRIMR7_27805	1	1	1
SRIMR7_06230	1	1	2	SRIMR7_28170	1	1	1
SRIMR7_06375	1	1	2	SRIMR7_28180	1	1	1
SRIMR7_06390	1	1	2	SRIMR7_28185	1	1	1
SRIMR7_06735	1	1	2	SRIMR7_28265	1	1	1
SRIMR7_07215	1	1	2	SRIMR7_28620	1	1	1
SRIMR7_07225	1	1	2	SRIMR7_28630	1	1	1
SRIMR7_07230	1	1	2	SRIMR7_28795	1	1	1
SRIMR7_07380	1	1	2	SRIMR7_28900	1	1	1
SRIMR7_07795	1	1	2	SRIMR7_30070	1	1	1
SRIMR7_07925	1	1	2	SRIMR7_30080	1	1	1
SRIMR7_08140	1	2	2	SRIMR7_30085	1	1	1
SRIMR7_08540	1	2	2	SRIMR7_30415	1	1	1
SRIMR7_08835	1	1	1	SRIMR7_30820	1	1	1
SRIMR7_08950	1	1	1	SRIMR7_30825	1	1	1
SRIMR7_08965	1	1	1	SRIMR7_30830	1	1	1
SRIMR7_08975	1	1	1	SRIMR7_30975	1	1	1
SRIMR7_09750	1	1	1	SRIMR7_31105	1	1	1
SRIMR7_10025	1	1	1	SRIMR7_31425	1	1	1
SRIMR7_10030	1	1	1	SRIMR7_31430	1	1	1
SRIMR7_10780	1	1	1	SRIMR7_31435	1	1	1
SRIMR7_11575	1	1	1	SRIMR7_31450	1	1	1
SRIMR7_12260	1	1	1	SRIMR7_31460	1	1	1
SRIMR7_12275	1	1	1	SRIMR7_31465	1	1	1
SRIMR7_12355	1	1	1	SRIMR7_31470	1	1	1
SRIMR7_12435	1	1	1	SRIMR7_31475	1	1	1
SRIMR7_12505	1	1	1	SRIMR7_31480	1	1	1
SRIMR7_12515	1	1	1	SRIMR7_32025	1	1	1
SRIMR7_12720	1	1	1	SRIMR7_32870	1	1	1
SRIMR7_12920	1	1	1	SRIMR7_32880	1	1	1
SRIMR7_13195	1	1	1	SRIMR7_33195	1	1	1

SRIMR7_13285	1	1	1	SRIMR7_33295	1	1	1
SRIMR7_14050	1	1	1	SRIMR7_33660	1	1	1
SRIMR7_14055	1	1	1	SRIMR7_34075	1	1	1
SRIMR7_14170	1	1	1	SRIMR7_34480	1	1	1
SRIMR7_14185	1	1	1	SRIMR7_35385	1	1	1
SRIMR7_14190	1	1	1	SRIMR7_35410	1	1	1
SRIMR7_14680	1	1	1	SRIMR7_35475	1	1	1
SRIMR7_14740	1	1	1	SRIMR7_35990	1	1	1
SRIMR7_15080	1	1	1	SRIMR7_36105	1	1	1
SRIMR7_15085	1	1	1	SRIMR7_36875	1	1	1
SRIMR7_15090	1	1	1	SRIMR7_36930	1	1	1
SRIMR7_15095	1	1	1	SRIMR7_36935	1	1	1
SRIMR7_15400	1	1	1	SRIMR7_37900	1	1	1
SRIMR7_15450	1	1	1	SRIMR7_37925	1	1	1
SRIMR7_15455	1	1	1	SRIMR7_38290	1	1	1

Table S2. BlastP identification of morphology regulators and their expression levels in *S. rimosus* HP126 and *S. rimosus* HP126 Δv3, compared to the wildtype as a control.

Locus tag	Annotation	Homolog and identity [%]	*S. rimosus* HP126	*S. rimosus* HP126 Δv3
*SRIMR7*_32110	Hypothetical protein	Chaplin, SRIM	-4.2	-1.8
*SRIMR7*_32115	Hypothetical protein	Chaplin, SRIM	-4.8	0
*SRIMR7*_18635	Hypothetical protein	Chaplin, SRIM,	3.9	-2.8
*SRIMR7*_33170	Hypothetical protein	Chaplin, SRIM	-3.2	-2.8
*SRIMR7*_04185	Hypothetical protein	Chaplin, SRIM	deleted	
*SRIMR7*_02675	Hypothetical protein	Chaplin, SRIM	1.8	-1.8
*SRIMR7*_38650	Hypothetical protein	Chaplin, SLAV, 46.25	3.2	-3.2
*SRIMR7*_34105	Sporulation and cell division protein	*ssgA*, SALB, 97.81	2.1	0
*SRIMR7*_06305	Sporulation and cell division protein	*ssgA*, SRIM	-1.0	1.1
*SRIMR7*_34105	Sporulation and cell division protein	*ssgA*, SCO, 43.9	-1.5	2.9
*SRIMR7*_02540	Major membrane protein I	Cyclic nucleotide-binding domain-containing protein, SRIM	-3.0	-5.8
*SRIMR7*_04760	Major membrane protein II	Cyclic nucleotide-binding domain-containing protein, SRIM	-2.9	-2.9
*SRIMR7*_04765	Major membrane protein III	Cyclic nucleotide-binding domain-containing protein, SRIM	-3.6	-2.7
*SRIMR7*_27140	Major membrane protein	Cyclic nucleotide-binding domain-containing protein, SRIM	-4.8	0
*SRIMR7*_10240	Bifunctional (p)ppGpp synthase/hydrolase, *relA*	Bifunctional (p)ppGpp synthase/hydrolase. SCO, 68.62	-1.4	0
*SRIMR7*_35510	Hypothetical protein	*bldB*, SALB, 46.1	1.1	-1.1
*SRIMR7*_12080	Transglutaminase-activating metalloprotease	Metalloprotease, SALB, 62.7	0	-1.6
*SRIMR7*_12085	Transglutaminase-activating metalloprotease	Metalloprotease, SALB, 60.55	1.3	-1.3
*SRIMR7*_21445	RNA polymerase sigma factor, sigX	*bldN*, SALB, 84.3	-1.8	-2.55

SRIM=*S. rimosus*; SLAV=*S. lavendofoliae*, SALB=*S. albus*; SCO= *S. coelicolor*

References

Gläser, L., Kuhl, M., Stegmuller, J., Ruckert, C., Myronovskyi, M., Kalinowski, J., et al. (2021) Superior production of heavy pamamycin derivatives using a bkdR deletion mutant of Streptomyces albus J1074/R2, *Microb Cell Fact* **20**: 111.

Kohlstedt, M., Sappa, P.K., Meyer, H., Maass, S., Zaprasis, A., Hoffmann, T., et al. (2014) Adaptation of *Bacillus subtilis* carbon core metabolism to simultaneous nutrient limitation and osmotic challenge: a multi-omics perspective, *Environ Microbiol* **16**: 1898-1917.

Kolde, R. (2019) Pheatmap: Pretty Heatmaps. R Package Version 1.0.12, https://CRANR-project.org/package=pheatmap.

Love, M.I., Huber, W., and Anders, S. (2014) Moderated estimation of fold change and dispersion for RNA-seq data with DESeq2, *Genome Biol* **15**: 550.

Love, M.I., Huber, W., and Anders, S. (2014) Moderated estimation of fold change and dispersion for RNA-seq data with DESeq2, *Genome Biol* **15**: 1-21.

McDowall, K.J., Thamchaipenet, A., and Hunter, I.S. (1999) Phosphate control of oxytetracycline production by Streptomyces rimosus is at the level of transcription from promoters overlapped by tandem repeats similar to those of the DNA-binding sites of the OmpR family, *J Bacteriol* **181**: 3025-3032.

Wickham, H., Chang, W., and Wickham, M.H. (2016) Package ‘ggplot2’, *Create Elegant Data Visualisations Using the Grammar of Graphics. Version* **2**: 1-189.
